# Integrative transcriptomics and proteomics profiling of *Arabidopsis thaliana* elucidates novel mechanisms underlying spaceflight adaptation

**DOI:** 10.3389/fpls.2023.1260429

**Published:** 2023-11-27

**Authors:** Gbolaga O. Olanrewaju, Natasha J. Haveman, Michael J. Naldrett, Anna-Lisa Paul, Robert J. Ferl, Sarah E. Wyatt

**Affiliations:** ^1^ Interdisciplinary Molecular and Cellular Biology Program, Ohio University, Athens, OH, United States; ^2^ Department of Environmental and Plant Biology, Ohio University Athens, OH, United States; ^3^ NASA Utilization & Life Sciences Office (UB-A), Kennedy Space Center, Merritt Island, FL, United States; ^4^ Proteomics & Metabolomics Facility, Nebraska Center for Biotechnology, University of Nebraska–Lincoln, Lincoln, NE, United States; ^5^ Department of Horticultural Sciences, University of Florida, Gainesville, FL, United States; ^6^ Interdisciplinary Center for Biotechnology Research, University of Florida, Gainesville, FL, United States; ^7^ Office of Research, University of Florida, Gainesville, FL, United States

**Keywords:** spaceflight, gravitropism, Arabidopsis, proteomics, RNAseq, TMT, BRIC LED, International Space Station

## Abstract

Spaceflight presents a unique environment with complex stressors, including microgravity and radiation, that can influence plant physiology at molecular levels. Combining transcriptomics and proteomics approaches, this research gives insights into the coordination of transcriptome and proteome in Arabidopsis’ molecular and physiological responses to Spaceflight environmental stress. Arabidopsis seedlings were germinated and grown in microgravity (µ*g*) aboard the International Space Station (ISS) in NASA Biological Research in Canisters – Light Emitting Diode (BRIC LED) hardware, with the ground control established on Earth. At 10 days old, seedlings were frozen in RNA-later and returned to Earth. RNA-seq transcriptomics and TMT-labeled LC-MS/MS proteomic analysis of cellular fractionates from the plant tissues suggest the alteration of the photosynthetic machinery (PSII and PSI) in spaceflight, with the plant shifting photosystem core-regulatory proteins in an organ-specific manner to adapt to the microgravity environment. An overview of the ribosome, spliceosome, and proteasome activities in spaceflight revealed a significant abundance of transcripts and proteins involved in protease binding, nuclease activities, and mRNA binding in spaceflight, while those involved in tRNA binding, exoribonuclease activity, and RNA helicase activity were less abundant in spaceflight. CELLULOSE SYNTHASES (CESA1, CESA3, CESA5, CESA7) and CELLULOSE-LIKE PROTEINS (CSLE1, CSLG3), involved in cellulose deposition and TUBULIN COFACTOR B (TFCB) had reduced abundance in spaceflight. This contrasts with the increased expression of UDP-ARABINOPYRANOSE MUTASEs, involved in the biosynthesis of cell wall non-cellulosic polysaccharides, in spaceflight. Both transcripts and proteome suggested an altered polar auxin redistribution, lipid, and ionic intracellular transportation in spaceflight. Analyses also suggest an increased metabolic energy requirement for plants in Space than on Earth, hence, the activation of several shunt metabolic pathways. This study provides novel insights, based on integrated RNA and protein data, on how plants adapt to the spaceflight environment and it is a step further at achieving sustainable crop production in Space.

## 1 Introduction

Numerous plant spaceflight studies have long focused on unraveling the complex physiological changes plants undergo in the spaceflight environment. In their diversity, these studies have accumulated an extensive array of phenotypic characterizations and uncovered some of the fundamental concepts related to physiological adaptation in space (Kordyum and Hasenstein, 2021). However, the traditional approaches have recently been complemented and in many ways superseded by the advent of genomic, transcriptomic, and proteomic methods, setting the stage for high throughput analyses that investigates the effects of spaceflight on plants from a molecular and functional perspective (Hughes and Kiss, 2022). This allows the physiological effects of the spaceflight environment to be characterized beyond phenotyping and extensively mapped out along broader biochemical pathways and cryptic signaling molecular cascades. While some remarkable advancements in genomic and transcriptomics studies of plants in spaceflight have been widely recorded (Ferl et al., 2002; Paul et al., 2012; Barker et al., 2023), such studies have largely been conducted in isolation, independent of proteomics. Unlike genomic and transcriptomic studies, proteomics studies face certain limitations in the spaceflight context, predominantly due to the limited availability of plant tissues for analysis. As a result, comprehensive spaceflight proteomics studies conducted on the whole Arabidopsis plant have been relatively few, with only three reported so far (Mazars et al., 2014; Ferl et al., 2015; Barker et al., 2020; Kruse et al., 2020).

Mazars et al. (2014) conducted the first true proteomics-scale study of Arabidopsis microsome-associated membrane proteins on the ISS followed by Hausmann et al. (2014) study on the proteome of Arabidopsis cell cultures in parabolic flights and Zhang et al. (2015) proteomic study on Arabidopsis callus aboard the Chinese SZ-8 spacecraft in spaceflight. Ferl et al. (2015) comparison of the transcriptome and protein abundances between the leaf and root of Arabidopsis in spaceflight represented the first use of a combined transcriptomics-proteomics investigative approach for whole plant spaceflight experiment, followed by a similar but more in-depth approach by Kruse et al. (2020) where they revealed novel regulatory responses of Arabidopsis seedlings in spaceflight. Reports from these two multi-omics studies indicated that the true power of omics approaches becomes apparent when they are combined, providing a comprehensive view of how gene expression and protein synthesis respond to the unique conditions of spaceflight. Likewise, the reported low correlation between transcripts and proteome of Arabidopsis grown in spaceflight and spaceflight hardware (Basu et al., 2017; Kruse et al., 2017; Kruse et al., 2020) reinforced the benefits of a multi-omics approach.

Studying plants’ adaptation to the spaceflight environment usually involves plants being flown to the ISS in designated hardware, whose designs and subsequent improvements are often based on the objectives of the experiment at hand (Kanapskyte et al., 2021; Nicholson et al., 2021). One such hardware is the Biological Research In Canister – Light Emitting Diode (BRIC LED). BRIC LED hardware is designed to enhance the capabilities of existing BRIC Petri Dish Fixation Units (PDFU) by the incorporation of a customizable discreet lighting system. Hence, biological organisms growing in the BRIC LED hardware are illuminated by LEDs with options of four different wavelengths (blue, red, far-red, and white) (Howard and Caron, 2016). Spaceflight experiments in the BRIC LED hardware expose plants to light cues in microgravity, hence closely simulating a natural environment where plants have to simultaneously integrate light and gravitational signaling. The incorporation of weightlessness and light signaling is expected to elicit several physiological and molecular responses which are distinct from other hardware without a light source (Stankovic, 2018), with the responses often novel and a function of cross-talks among various signaling pathways rather than an individual signal transduction pathway. Tightly regulated by complex cross-signaling pathways, the responses are usually specific to each plant organ and most times, tissues (Wolinska and Berens, 2019). Transcripts and proteomes encoding these stress response pathways are usually localized to a particular intracellular or extracellular region, further reinforcing the idea that studies on plant stress response should be focused on finer resolutions of organs, tissues, cells, and organelles. Several studies have identified that the plant root and shoot respond in an organ-specific manner to gravity (Paul et al., 2013; Vandenbrink et al., 2014; Lamers et al., 2020) and other environmental stresses (Paponov et al., 2021). Hence, resolving plant proteomic responses to the spaceflight environment on an organ-specific level is a step further in understanding stress response signaling pathways and the molecular adaptations of each plant organ to the spaceflight environment and how such adaptation mechanisms can be manipulated for improved plant production in space and on earth.

Therefore, building upon these previous achievements in plant multi-omics research in spaceflight, we took a significant step forward by integrating both proteomics and transcriptomics approaches to understand the response of Arabidopsis plants to the spaceflight environment. Through increased coverage and finer spatial proteomic resolution, we assessed the effects of the earth’s low orbit spaceflight environment aboard the ISS on the shoot and root of the Arabidopsis seedlings. The seedlings were grown in the NASA BRIC LED hardware, hence we provided robust insight into the plant transcriptome landscape and organ-specific proteomic responses of Arabidopsis to the spaceflight environment, while at the same time conducting a hardware verification test for the novel BRIC LED hardware.

## 2 Materials and methods

### 2.1 Plant material and spaceflight growth conditions

*Arabidopsis thaliana* L., ecotype Columbia-0 seeds were surface sterilized with 30% (v/v) bleach, and 0.1% (v/v) Tween-20 for 2 mins, washed repeatedly with sterile water (5×) and dried on round filter paper. Approximately 30 seeds were plated on each 60mm round petri plate containing 6.7ml of 0.5× + Benomyl Murashige and Skoog (MS) growth media with Gamborg’s B5 vitamins, 0.5% (w/v) sucrose and 0.75% (w/v) phytagel, and cold stratified for 16h. Seeded plates were loaded in a horizontal orientation, with the face of the growth media parallel to the ground in the BRIC LED hardware petri dish fixation units (PDFUs). Each hardware canister holds 6 PDFUs, with each PDFU holding a 60mm petri plate. This configuration was replicated 6 times, making a total of 36 seeded 60mm plates loaded aboard ISS. All BRICs were kept in cold storage (4°C) prior to the spaceflight launch. The experiment was flown on the SpaceX CRS-13 mission launched aboard the Falcon 9 Full Thrust rocket on December 15, 2017. The dormant Arabidopsis seeds were activated with light and placed at ambient temperature aboard the ISS, with a light wavelength composition of 85% red, 15% blue, and 60 µmol m^−2^ s^−1^ intensity. The plants were grown for 10 days with a 4-hour/2-hour light-dark cycle, prior to actuation of the PDFUs, releasing about 6–7ml RNAlater into the petri plates for seedling fixation. The seedlings were then incubated in Invitrogen RNAlater for 12 hours after which they were frozen aboard the ISS MELFI at −80°C until returned to Earth on May 5, 2018, aboard the SpaceX CRS-14 cargo dragon. A similar experimental setup was established on the ground, for same duration, at the Kennedy Space Center in Florida in the Space Station Environmental Simulator (ISSES) chamber, USA. The samples were transported on dry ice from the dragon capsule to the receiving laboratories. Frozen plates containing both the spaceflight and ground samples were packed in dry ice and transported to Ohio University where they were stored at −80°C prior to protein extraction and processing while another group was also sent to the University of Florida, Gainesville for RNA extraction, library preparation and sequencing.

### 2.2 RNA extraction and sequencing

For each condition (Spaceflight and Ground), four individual plants from each replicate were pooled for RNA extraction. Individual plants were first dissected into leaves, hypocotyls, and root tissues under an Olympus dissecting microscope (Olympus, Japan). The intervening hypocotyls were set aside and not processed for downstream analyses. Leaf tissues without their hypocotyl are referred to herein as shoots and are the only ones processed for RNA analysis. RNA extraction was performed using the Qiagen RNeasy Plant Mini Kit (Qiagen, Germany) according to the manufacturer’s guidelines. RNA concentration was assessed using the Qubit 2.0 Fluorometer (ThermoFisher Scientific, USA), and RNA quality was determined on the Agilent 2100 Bioanalyzer (Agilent Technologies Inc., USA). For all samples, the RNA integrity number (RIN) was more than 6.

RNA-Sequencing Library Preparation was performed at the University of Florida Interdisciplinary Center for Biotechnology Research (ICBR) Gene Expression Core (https://biotech.ufl.edu/gene-expression-genotyping/ RRID: SCR_019145). Here, 100 ng of total RNA was used for mRNA isolation using the NEBNext Poly(A) mRNA Magnetic Isolation Module (New England Biolabs, catalog #E7490). Next, RNA library construction was done with the NEBNext Ultra II Directional RNA Library Prep Kit (New England Biolabs, catalog #E7760) according to the manufacturer’s user guide. Briefly, RNA was fragmented in NEBNext First Strand Synthesis Buffer via incubation at 94°C for the desired time. This step was followed by first-strand cDNA synthesis using reverse transcriptase and random hexamer primer. Synthesis of ds-cDNA was performed using the 2nd strand master mix provided in the kit, followed by end-repair and adaptor ligation. At this point, Illumina adaptors were ligated to the sample. Finally, each library (uniquely barcoded) was enriched by 12 cycles of amplification, and purified with Agencourt AMPure beads (Beckman Coulter, catalog #A63881). The 12 barcoded libraries were sized on the Bioanalyzer and quantified with the Qubit^®^ 2.0 Fluorometer. Finally, these 20 individual libraries were pooled in equimolar concentration.

The Illumina NovaSeq 6000 was used to sequence the libraries for 2 × 150 cycles. Sequencing was performed at the ICBR NextGen Sequencing (https://biotech.ufl.edu/next-gen-dna/, RRID: SCR_019152). Normalized libraries were submitted to the “Free Adapter Blocking Reagent” protocol (FAB, catalog #20024145) to minimize the presence of adaptor-dimers and index hopping rates. The library pool was diluted to 0.8 nM and sequenced on one S4 flow cell lane (2 × 150 cycles) of the Illumina NovaSeq6000. The instrument’s computer utilized the NovaSeq Control Software v1.6. Cluster and SBS consumables were v1.5. The final loading concentration of the library was 120 pM with 1% PhiX (v/v) spike-in control. One lane generated 2.5–3 billion paired-end reads (~950Gb) with an average Q30%≥92.5% and Cluster PF=85.4%. FastQ files were generated using the BCL2fastQ function in the Illumina BaseSpace portal. One NovaSeq S4, 2 × 150 cycles lane resulted in an average of 50 million demultiplexed, paired-end reads when sequencing a pool of 48 samples.

### 2.3 Protein extraction, sample processing and LC-MS/MS

Seedlings from spaceflight and ground controls were divided into root and shoot for soluble and membrane protein extractions according to a protocol adapted from Basu et al. (2015). Approximately 0.1g of tissue per replicate was crushed in liquid nitrogen and vortexed thoroughly in the extraction buffer. The samples were subsequently centrifuged at 8000g and 100,000g to isolate membrane protein. Both the precipitated soluble protein stored in methanol and the membrane protein in microsome resuspension buffer were sent to the Proteomics and Metabolomics Facility, at the University of Nebraska, Lincoln for 6-plex TMT labeling and mass spectrometric analysis (**Supplementary protocol**).

### 2.4 Data analysis

RNA read pairs for each biological replicate for the flight and ground control samples were checked for sequence quality using the FastQC, trimmed using the TrimGalore, and aligned to TAIR10 assembly using the Spliced Transcripts Alignment to a Reference (STAR) (Kruse et al., 2020). Determination of gene-level read counts was accomplished using the Feature-count tool while the differential expression was determined using the negative binomial generalized linear model likelihood tests with the DESeq2 package in R. LC-MS/MS peptide data were analyzed using Proteome Discoverer 2.4 software (ThermoFisher Scientific, USA) using the Sequest HT search engine. Peptides were validated by Percolator with a 0.01 posterior error probability (PEP) threshold (Rioseras et al., 2018). The data were searched using a decoy database to set the false discovery rate to 1% (high confidence). Protein quantification was processed using the co-isolation threshold set to 50% and the average S/N to 10, and peptides were quantified using the peak intensity of the reporter ions in the MS3 spectra. The peak abundance was normalized for differences in sample loading using total peptide amount where the peptide group abundances are summed for each sample and the maximum sum across all runs is determined. The protein ratios and log2 fold changes (L_2_FC) were calculated from the grouped protein abundances. RNA transcripts and Proteins with adjusted *p*-value <0.05 were considered statistically significant while thresholds of L_2_FC≥1 or ≤−1 and L_2_FC≥0.2 or ≤−0.2 were established for the RNA and protein respectively to decide differential abundance between spaceflight and earth.

### 2.5 Analysis of the gene ontology enrichment

Analysis of the gene ontology (GO) enrichment was performed using the ShinyGO V0.77 (based on Ensembl Release 107 with revision, archived on April 4, 2022) (Ge et al., 2020) and confirmed using the DAVID database (Sherman et al., 2022). Only differentially abundant (*p*<0.05) RNA and proteins with significant abundance in spaceflight (L_2_FC≥1 and L_2_FC≥0.2 respectively) or significant abundance on earth (L_2_FC≤−1 and L_2_FC≤−0.2 respectively) were analyzed for fold enrichment. The GO term filter condition was set at a false discovery rate (FDR) of 0.01 and *p*-values (ratio of total genes involved in a pathway found in the dataset to total genes in the pathway) were adjusted by the Benjamini–Hochberg (B–H) method to reduce false positives. Hence, poorly represented annotated terms and overly broad terms in the protein dataset were eliminated. Likewise, a comparison was drawn between the araPath enrichment terms (Lai et al., 2012) and STRINGdb (www.https://string-db.org/) terms in the dataset to ensure we get an accurate overview of the GO enrichment. In cases where we have similar enrichment terms, only the most informative terms, with fewer similarities and the highest enrichment value were retained. Visual representations of the resulting enrichment terms were created using the graph tools on ShinyGO and the “ggplot”, “GOsummaries” and “Heatmap” packages in RStudio (Posit team, 2023).

### 2.6 Confirmation of subcellular localization

The subcellular locations of differentially abundant (*p*<0.05) RNA and proteins in spaceflight and on the ground were analyzed using the Subcellular Localization Database for Arabidopsis Proteins (SUBA4, http://suba.live, accessed May 2023). Specific protein consensus subcellular localizations were decided using the SUBAcon (Hooper et al., 2014), a consensus algorithm for unifying the subcellular localization data of the Arabidopsis proteome. The resulting subcellular localizations were plotted on a sunburst chart.

## 3 Results

### 3.1 Transcriptomics and proteomics recovery in the BRIC LED experiment

**Figure 1:**
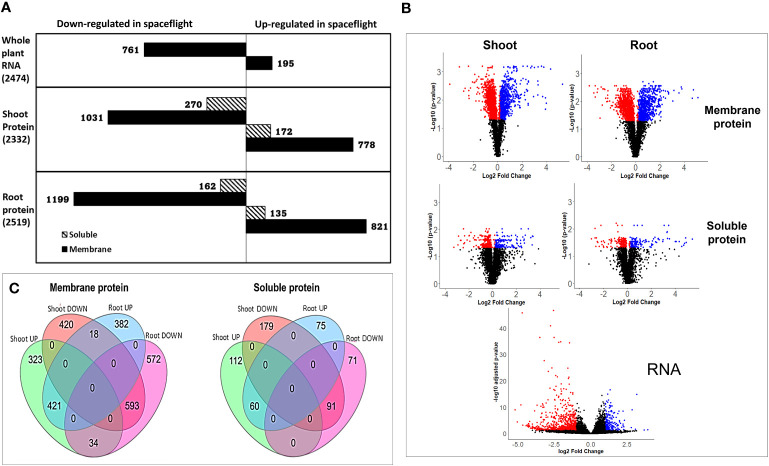
Transcriptome and proteome recovery in BRIC LED experiment. **(A)**. Bar plots of the RNA and protein abundance distribution in spaceflight and in ground control. On the protein plots, solid-colored bars represent the membrane proteins while hashed bars represent the soluble protein. The quantity of differentially abundant RNA and proteins (*p*<0.05) is indicated in parentheses. RNA and proteins with L_2_FC_RNA_≥1 and L_2_FC_protein_≥0.2 respectively were considered abundant in spaceflight while those with L_2_FC_RNA_≤−1 and L_2_FC_protein_≤−0.2 respectively were considered less abundant in space. **(B)**. Volcano plot of the total abundance and distribution of RNA and proteins between spaceflight and ground tissues. The negative log of the adjusted *p*-values was plotted on the y-axis against the normalized log2 fold change on the x-axis. Dots represent RNA and protein abundance with black dots representing non-significantly expressed proteins (*p>0.05* and those with L_2_FC not within the threshold), red dots represent less abundant RNA and proteins in spaceflight while blue dots represent proteins that were more abundant in spaceflight than on earth. **(C)**. Venn diagram of differential protein abundance in spaceflight. Combination of differential proteins more abundant in spaceflight and less abundant in spaceflight, as distinct to each organ and as shared by both organs. Shoot UP indicates shoot proteins more abundant in spaceflight, shoot DOWN indicates shoot proteins less abundant in spaceflight, Root UP indicates root proteins more abundant in spaceflight while root DOWN indicates root proteins less abundant in spaceflight.

The differential analysis of the transcriptome expressed between spaceflight and ground control Arabidopsis samples yielded 15,505 distinctly mapped RNA transcripts, with 2,474 being statistically significant at adjusted *p*<0.05. Of this, 195 RNA are upregulated in spaceflight at L_2_FC_RNA_≥1, while 761 are downregulated in spaceflight at L_2_FC_RNA_≤−1 (**Figure 1A**). Protein extraction from the different organs (root and shoot) and across the cellular fractions (soluble and membrane) led to the quantification, in the root, of 20,923 peptides mapped to 3973 membrane proteins and 18,636 peptides mapped to 3412 soluble proteins. Similar to this, in the shoot, 20,658 peptides were mapped to 3965 membrane proteins and 20,091 peptides were mapped to 3537 soluble proteins. Only proteins with a FDR of 0.01 and a minimum of two uniquely mapped non-nested peptides (amino acid length >6 were retained. Proteins with B–H adjusted *p*≤0.05 were identified as statistically significant in the dataset. A total of 2187 membrane proteins and 332 soluble proteins were statistically significant in the root, while 1856 membrane proteins and 476 soluble proteins were statistically significant in the shoot. At the decision thresholds of L_2_FC_protein_≥0.2 and L_2_FC_protein_≤−0.2, 821 membrane proteins and 135 soluble proteins in the root were more abundant in spaceflight, whereas 1199 membrane proteins and 162 soluble proteins were less abundant in spaceflight (**Figure 1A**). Likewise, in the shoot, 1031 and 270 membrane and soluble proteins respectively were less abundant in spaceflight compared to ground control, while 778 membrane proteins and 172 soluble proteins were more abundant in spaceflight compared to ground control (**Figure 1A**).

The volcano plot (**Figure 1B**) of the total mapped RNA and protein across the organs and cell fractions gives a broader view of the pattern of abundance of transcriptome and proteome in spaceflight. Applying the widely accepted threshold for RNA tier II analysis, L_2_FC_RNA_≥1 or L_2_FC_RNA_≤−1, reduces false positives in interpreting the biological significance of the differentially expressed transcripts (McDermaid et al., 2019). However, this also led to the exclusion of over 61% of our total statistically significant transcripts (**Figure 1B**), with the possibility of the exclusion of some transcripts with biological significance. Membrane proteins were more differentially abundant (*p*<0.05) and highly perturbed in spaceflight compared to the soluble proteins. Membrane proteins in the root had the largest abundance and fold enrichment, followed by the membrane and soluble proteins in the shoots respectively, while soluble proteins in the root were the least quantified and least differentially abundant (**Figure 1B**).

Several studies (Sarasketa et al., 2016; Paponov et al., 2021) have identified specificity in the proteomic profile of root and shoot in response to various environmental perturbations such as magnetic field, hormonal homeostasis, and gravity. This study further provides insight into how the specific plant organs (root and shoot) respond to microgravity in spaceflight. The organ-targeted proteomic profiling in response to microgravity, in this study, revealed that roots and shoots adapt differently to the spaceflight environment, and at the same time, they share certain commonalities. Comparing root-specific protein abundance in spaceflight to Earth, 400 membrane proteins and 75 soluble proteins were more abundant in space, whereas 606 membrane proteins and 71 soluble proteins were less abundant in spaceflight (**Figure 1C**). For proteins specific to the shoot, 357 membrane, and 112 soluble proteins were more abundant in spaceflight, whereas 438 membrane and 179 soluble proteins were less abundant in spaceflight (**Figure 1C**). Common to both root and shoot, 421 membrane proteins and 60 soluble proteins were more abundant in spaceflight compared to on Earth. Likewise, the abundance of 593 membrane proteins and 91 soluble proteins was reduced in spaceflight across both organs. Interestingly, a comparison of the differentially abundant proteins shared by both organs revealed that 18 membrane proteins were more abundant in spaceflight in the root and less abundant in the shoot, while 34 membrane proteins were more abundant in spaceflight in the shoot and less abundant in the root (**Figure 1C**, **Table 1**). A GO enrichment and KEGG pathway analysis of the 34 membrane proteins with increased abundance in the shoot and decreased abundance in the root in spaceflight revealed that they were majorly involved in the photosynthetic pathway, carbon metabolism pathway, and amide biosynthetic process. Likewise, these proteins were majorly chloroplast localized and are enriched for glyceraldehyde 3-phosphate dehydrogenase (NAD+) phosphorylating activity, poly(U) RNA binding, and oxidoreductase activity (**Supplementary Figure 1**). KEGG pathway analysis of the 18 membrane proteins that were more abundant in spaceflight in the root and less abundant in the shoot did not indicate their involvements in a major metabolic pathway. However, some of these proteins were the heat shock proteins (HSP70, HSP90.4), host defense proteins such as the Importin (IMPA-4) and Pectinesterase inhibitor 17 (PME17), proteins involved in shunt energy processes such as the Transketolase (TLK2) and Electron Transfer flavoprotein (ETFQ). (**Table 1**).

**Table 1:** Proteins differentially expressed between the root and shoot.

TAIR ID	Protein name	Root		Shoot	
		L2FC	*p*-value	L2FC	*p*-value
Upregulated in shoot, downregulated in root					
AT1G04800	Glycine-rich protein	−0.34	0.029	0.97	0.04
AT1G13440	Glyceraldehyde-3-ph	−0.51	0.031	0.37	0.02
AT1G18080	Receptor for activated C kinase 1A	−0.23	0.036	0.26	0.03
AT1G21380	TOM1-like protein 3	−0.27	0.022	0.95	0.04
AT1G44446	Chlorophyllide a oxygenase	−0.64	0.02	0.41	0.05
AT1G79530	Glyceraldehyde-3-ph	−0.22	0.017	0.56	0.01
AT2G02050	NADH dehydrogenase [ubiquinone] 1 beta subcomplex subunit 7	−0.32	0.018	0.68	0.04
AT2G34420	Chlorophyll a–b binding protein	−0.39	0.04	3.04	0.01
AT2G34430	Chlorophyll a–b binding protein	−0.64	0.048	1.69	0.03
AT2G37040	Phenylalanine ammonia-lyase 1	−0.38	0.01	0.33	0.01
AT2G38040	Acetyl-coenzyme A carboxylase carboxyl transferase subunit alpha	−0.2	0.003	0.24	0.01
AT2G44120	60S ribosomal protein	−0.39	0.011	0.37	0.02
AT2G45180	Disease non-specific lipid transfer protein	−0.35	0.012	1.47	0.03
AT2G47510	Fumarate hydratase 1	−0.27	0.033	0.35	0.01
AT3G13460	Evolutionarily conserved C-terminal region 2	−0.21	0.039	0.48	0.02
AT3G19480	D-3-phosphoglycerate dehydrogenase	−0.27	0.02	0.44	0.02
AT3G25920	50S large ribosomal subunit	−0.34	0.011	0.82	0.01
AT3G50820	Oxygen-evolving enhancer protein 1–2	−0.41	0.014	0.72	0.01
AT3G60770	40S ribosomal subunit	−0.21	0.04	0.66	0.02
AT3G62530	ARM repeat superfamily protein	−0.34	0.004	0.69	0.01
AT4G04020	Probable plastid-lipid-associated protein 1	−0.33	0.012	0.4	0.03
AT4G11420	Eukaryotic translation initiation factor 3 subunit A	−0.24	0.007	0.54	0.04
AT4G29480	Mitochondrial ATP synthase subunit G protein	−0.33	0.015	1.19	0.02
AT4G30720	FAD/NAD(P)-binding oxidoreductase family protein	−0.3	0.009	0.53	0.01
AT4G34110	Polyadenylate-binding protein 2	−0.24	0.022	0.41	0.04
AT5G22880	Histone H2B.10	−0.27	0.017	0.25	0.04
AT5G46580	Pentatricopeptide repeat-containing protein At5g46580	−0.68	0.004	0.56	0.02
AT5G48030	Chaperone protein dnaJ GFA2	−0.55	0.013	0.96	0.03
AT5G48810	Cytochrome B5 isoform D	−0.32	0.021	1.38	0
AT5G53620	RNA polymerase II degradation factor	−0.37	0.037	0.48	0.04
AT5G64110	Peroxidase 70	−0.34	0.01	0.46	0
AT5G64860	4-alpha-glucanotransferase DPE1	−0.29	0.036	0.78	0.03
AT5G65010	Asparagine synthetase [glutamine-hydrolyzing] 2	−0.33	0.012	0.3	0.02
ATCG00790	50S large ribosomal subunit	−0.42	0.01	0.82	0.02
Downregulated in shoot, upregulated in root					
AT1G09270	Importin subunit alpha-4	0.25	0.01	-0.49	0.02
AT1G11660	Heat shock protein 70 (Hsp 70) family protein	0.2	0.039	-0.21	0.04
AT1G24100	UDP-Glucosyl transferase 74B1	0.66	0.013	-0.24	0.02
AT1G49730	Probable receptor-like protein kinase At1g49730	0.31	0.026	-1.4	0.01
AT1G55020	Linoleate 9S-lipoxygenase 1	0.32	0.017	-0.26	0.03
AT1G65820	Microsomal gluthatione s-transferase	0.31	0.017	-0.27	0.03
AT2G43400	Electron transfer flavoprotein-ubiquinone oxidoreductase	0.58	0.006	-0.6	0.04
AT2G45220	Probable pectinesterase/pectinesterase inhibitor 17	0.87	0.00	-0.28	0.04
AT2G45290	Transketolase-2	0.23	0.045	-0.81	0.03
AT3G22960	Plastidial pyruvate kinase 1	0.29	0.013	-0.45	0.05
AT4G25080	Magnesium protoporphyrin IX methyltransferase	0.24	0.035	-0.78	0.03
AT4G38690	PLC-like phosphodiesterases	0.28	0.017	-0.41	0.02
AT5G01750	Protein LURP-one-related 15	0.44	0.044	-0.29	0.02
AT5G03300	Adenosine kinase 2	0.45	0.035	-0.44	0.04
AT5G23575	Transmembrane CLPTM1 family protein	0.33	0.01	-0.38	0.01
AT5G56000	Heat shock protein 90-4	0.4	0.02	-0.41	0.04
AT5G56590	O-Glycosyl hydrolases family 17 protein	0.31	0.01	-0.36	0.01
AT5G59420	Oxysterol-binding protein-related protein 3C	0.35	0.003	-0.36	0.03

### 3.2 Subcellular distribution and abundance of protein in spaceflight

Several metabolic processes in cells, such as signal transduction, transport of molecules, cell division, cell death, etc., are tightly linked with the location of specific transcripts and proteins (Christopher et al., 2022). Transcripts are targeted toward different subcellular organelles in the plant cell based on the specific functions of encoded proteins. Assessing which of the targeted organelles have the highest amount of perturbed proteins gives insight into the metabolic process most affected by the spaceflight environment. Our expectation is that since transcripts encode proteins that are targeted toward different organelles, the pattern of subcellular localization of the RNA transcripts differentially expressed should be similar to that of proteins differentially expressed in spaceflight. To ascertain this, we compared transcripts and proteins with differential abundance in our spaceflight experiments against SUBA4, and results were subsequently fed into SUBAcon (Hooper et al., 2017) to recover more accurate proteome-wide subcellular localizations.

**Figure 2:**
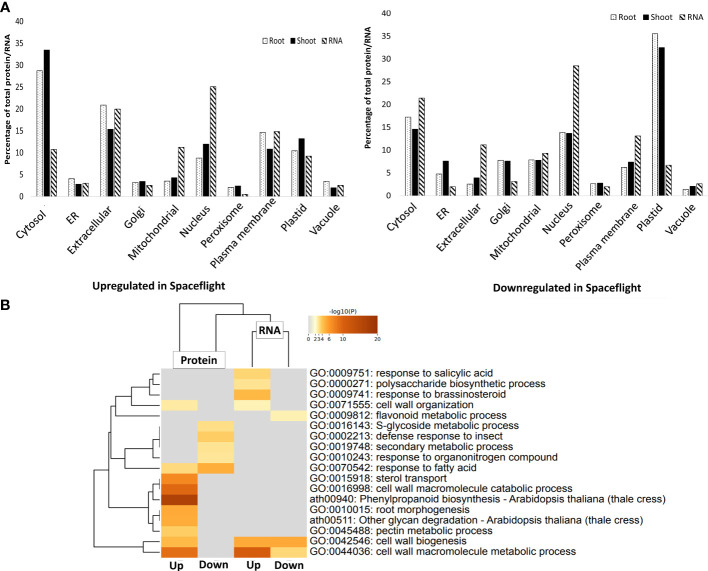
Subcellular localization of differentially abundant RNA and proteins in spaceflight. **(A)**. Bar chart comparing the percentage of RNA transcripts (hashed bars) and root (dotted bars) and shoot (black bars) proteins localized in each subcellular compartment. The subcellular localizations were identified using SUBA4 and localization consensus was determined using SUBAcon. **(B)**. GO enrichment heat map and hierarchical cluster tree of transcripts that encode extracellular matrix-targeted proteins and the proteins that are localized in the extracellular matrix. The color gradient of the heatmap indicates the significance of the genes and proteins in the enriched metabolic pathways. Labels with “Up” means upregulated in spaceflight and downregulated on Earth while labels with “Down” indicates downregulated in spaceflight and upregulated on Earth.

Transcripts encoding nucleus-targeted proteins constituted the largest percentage of the transcripts upregulated (25%) and downregulated (28.5%) in spaceflight (**Figure 2A**). KEGG pathway analysis of the nucleus-targeted transcripts upregulated in spaceflight indicated that they are majorly involved in plant hormone signal transduction pathways which are tryptophan metabolism, zeatin biosynthesis, diterpenoid biosynthesis, and phenylalanine metabolism (**Supplementary Figure 2A**). Transcripts encoding nucleus-targeted proteins downregulated in spaceflight are involved in protein processing in the endoplasmic reticulum and ribosome pathway, with many being part of the ubiquitin ligase complex and the ER-associated degradation complex (ERAD) (**Supplementary Figure 2B**). Another interesting group of transcripts are those encoding proteins targeted towards extracellular matrix, being 20% of the total upregulated RNA and 11.1% of the total downregulated RNA. Upregulated transcripts encoding extracellular-matrix targeted proteins are enriched in cell wall biogenesis, cell wall macromolecule metabolic process, and flavonoid metabolic process, while the downregulated ones are uniquely enriched in pathways involved in response to salicylic acid, polysaccharide biosynthetic process, response to brassinosteroid and cell wall organization (**Figure 2B**). The metabolic pathways enriched were significantly different in the extracellular protein dataset in comparison with the RNA transcriptome (**Figure 2B**).

In the root, plastid localized proteins constituted 35.5% of soluble and membrane proteins with reduced abundance in spaceflight, while they constituted 10.3% of proteins more abundant in spaceflight than on earth (**Figure 2A**). Enrichment analysis of root plastid localized proteins that were differentially abundant in spaceflight indicated that proteins involved in plastid movements, organization of plastid membranes, and light harvesting were less abundant in spaceflight than on earth, while those involved in valine biosynthetic pathways, reductive pentose-phosphate cycle, and the dark reaction of photosynthesis were more abundant in spaceflight. The valine biosynthetic pathway is intertwined with the isoleucine biosynthetic pathway, and they both inhibit the growth of shoots (Cao et al., 2019) and increase root formation. Notably, 154 membrane and 51 soluble extracellular localized proteins were abundant in spaceflight in the root and they are majorly involved in glycan degradation, phenylpropanoid biosynthesis, and pentose-glucuronate interconversion. This is indicative of increased restructuring of the root cell wall polysaccharides in spaceflight.

The distribution of protein localization in the shoot is similar to the root, with plastid localized proteins constituting 32.4% of proteins less abundant in spaceflight, while cytosolic protein constituted 33.4% of the proteins more abundant in spaceflight (**Figure 2A**). However, enrichment analysis of shoot plastid localized proteins indicated high enrichment of proteins involved in plastid transcription and organization, chlorophyll metabolism, and photosynthesis. Contrary to the root, 83 membrane and 63 soluble extracellular proteins were more abundant in spaceflight and they are highly enriched in pathways for cellular oxidation regulation and negative regulation of peptidase activities. Extracellular proteins less abundant in spaceflight are enriched in pathways responsible for plant defense. An interesting observation as regards the comparative subcellular localization of transcriptome and root and shoot proteome in spaceflight is that most downregulated RNA in spaceflight encode proteins targeted back to the nucleus whereas most downregulated proteins in spaceflight are plastids localized. Cytosolic proteins are most upregulated in spaceflight, whereas transcripts encoding nucleus-targeted proteins are most upregulated (**Figure 2A**).

### 3.3 Enrichment analysis of the gene ontology terms

**Figure 3:**
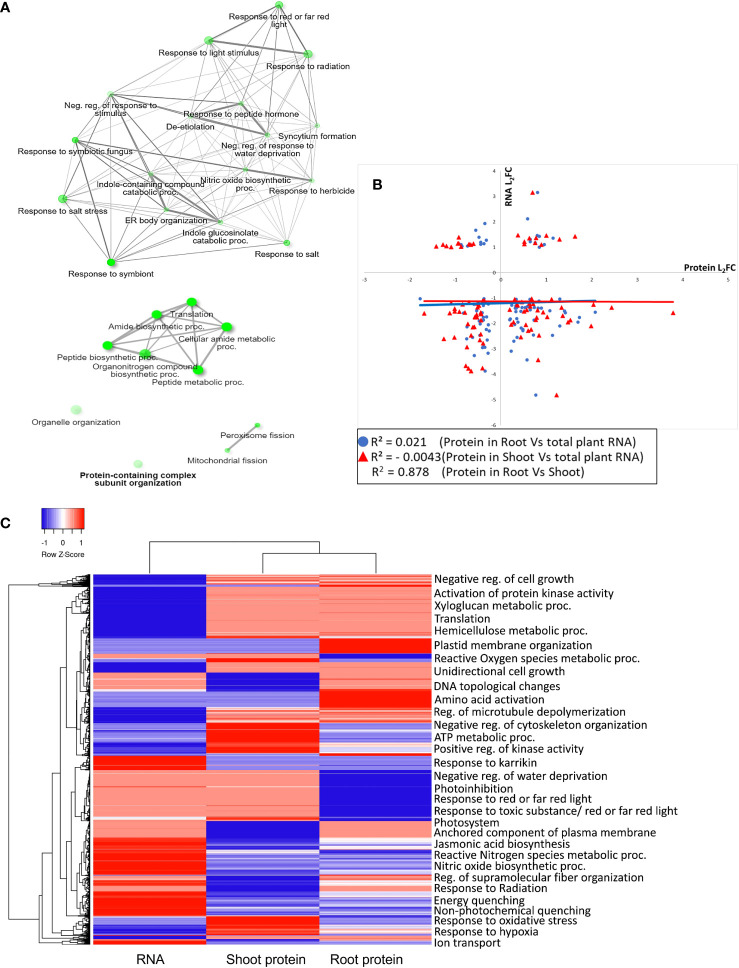
Gene ontology enrichment of RNA and proteins differentially expressed in spaceflight **(A)**. Network interaction map of RNA and proteins with a similar pattern of expression in spaceflight. Upregulated transcripts with upregulated proteins are above, while downregulated transcripts with downregulated proteins are below. The node sizes indicate the number of genes enriched in that pathway while two pathways (nodes) are connected only if they share a minimum of 20% genes. GO terms enrichment analyses were conducted in ShinyGO 0.77 (GO terms were accessed in June 2023). **(B)**. Correlation graph of the log2 fold change of shoot and root proteins with total RNA transcripts. The correlations were plotted separately with black triangles representing the correlation of the log2 fold change of proteins in the root and the log2 fold change of the plant total RNA (root vs total RNA) with a correlation coefficient, *r^2^=*0.021, and circles representing the correlation of the log2 fold change of proteins in the shoot and the log2 fold change of the plant total RNA (shoot vs total RNA) with a correlation coefficient*, r^2^* =−0.004. The plot of the correlation of the log2 fold change of shared proteins in the root and shoot (Root vs Shoot) is not shown on the graph. However, its correlation coefficient (*r^2^=*0.878) is displayed. **(C)**. Heatmap of the GO enrichment of gene expression patterns in RNA, shoot, and root proteins. The corresponding GO terms are listed to the right of the heat map. The color gradients represent the Z-score calculated from the L_2_FC, with blue as downregulated and red as upregulated. Both row and column hierarchical clusters indicate the similarities of the categories.

Creating a functional profile of transcriptome and proteome abundance in spaceflight elucidates various underlying biological, cellular and molecular processes affected by the harsh spaceflight environmental conditions such as microgravity, ionizing radiation and so on. Insight into the biological roles of these RNAs and proteins was acquired through enrichment analysis of the protein ontologies. A common feature in spaceflight, across the transcriptome and proteome landscapes, is the upregulation and enrichment of stress response proteins, especially those involved in response to light stimulus, radiation, oxygen, and water deprivation (**Figure 3A**). Enriched among the downregulated RNA and proteins are those involved in translation, amide biosynthetic process, mitochondrial and peroxisome fission (**Figure 3A**). A correlation plot of the expression (log2 fold change) of transcripts and the shoot and root proteins indicated very low gene expression correlation between shoot proteins and transcripts (*r*=−0.004) and between root proteins and transcripts (*r*=0.021). However, protein expression between the shoot and root is positively correlated (*r*=0.878) (**Figure 3B**). An overview of the gene ontology of the gene expression pattern between the transcripts and shoot and root proteins is represented by a heat map (**Figure 3C**).

Membrane and soluble proteins involved in the activation of protein kinase activity, regulation of microtubule depolymerization, and cellular detoxification were abundant and notably enriched in root and shoot (**Figure 3C**). Another important observation among proteins less abundant in spaceflight is the enrichment of transport proteins, cargo sorting proteins and vesicle loading proteins (**Supplementary Table A**). Proteins involved in the negative regulation of the cytoskeleton were abundant and highly enriched in spaceflight in both organs (**Supplementary Figures 3, 4**). It is unclear whether this was a response to spaceflight or a regulator of the response.

Specific to the root, proteins involved in DNA topological change, amino acid activation, and regulation of fiber organization were enriched among proteins more abundant in spaceflight while those involved in photoinhibition, cytoskeleton organization, and reactive oxygen species metabolic process were enriched among proteins less abundant in spaceflight (**Figure 3C**). Soluble proteins involved in mitochondrial elongation and the glyoxylate cycle were highly enriched in spaceflight (**Supplementary Figure 5**) suggesting the compensation of photosynthetic ATP production deficit by increasing metabolic fluxes through the glyoxylate cycle. Further analysis of the photosystems’ proteome supports this suggestion. In the shoot, a large proportion of the enriched proteins are involved in nucleotide metabolic processes, protein synthesis, and localization to several destinations (**Supplementary Figure 5**).

### 3.4 Alterations to the photosystems transcriptome and proteome in the spaceflight environment

**Figure 4:**
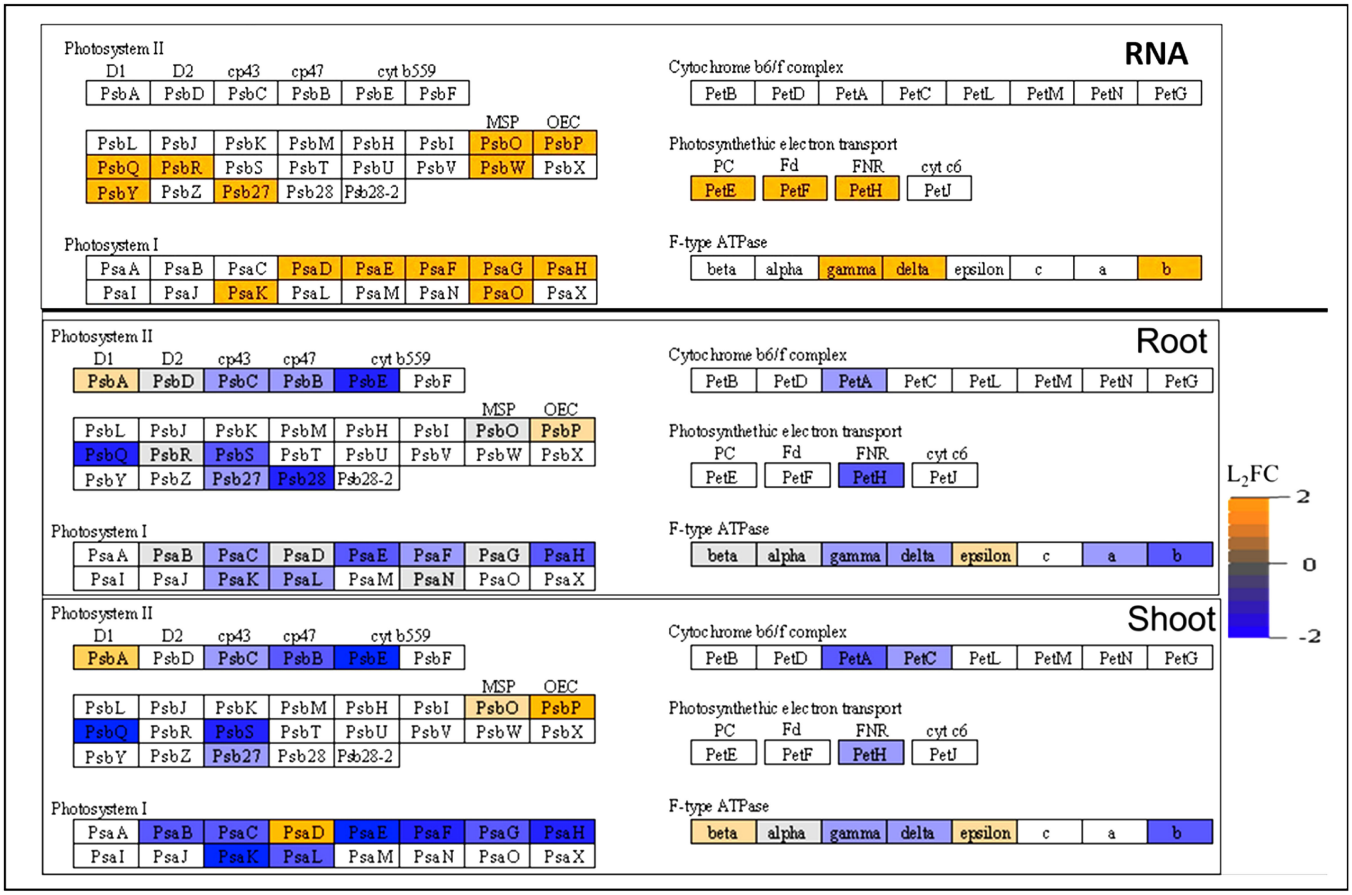
Abundance of photosystem RNA transcripts and proteins in spaceflight. The differential abundance of photosynthetic RNA transcript (top) proteins in the root (middle) and shoot (bottom) as determined by their Log2 fold change. The orange color indicates RNA transcripts and proteins that are more abundant in spaceflight, while the blue color indicates RNA transcripts and proteins that are less abundant in spaceflight. Uncolored boxes indicate RNA transcripts and proteins that were not significantly abundant in spaceflight and on Earth. This figure was developed by the KEGG path view. Supporting data can be found in the **Supplementary Table B**.

The presence of customizable discrete lighting in the BRIC LED hardware led to the enrichment of several RNA transcripts and proteins involved in the photosynthetic pathways (**Figure 4**, **Supplementary Table B**). Interestingly, all the photosystem RNA transcripts differentially expressed in spaceflight were upregulated, whereas most of them became less abundant in both the shoot and root proteomes (**Figure 4**). This is indicative of post-transcriptional regulation of the photosystem transcripts suggestive of a suppressive adaptation mechanism to reduce light harvesting ability of the photosystems.

Common in both organs and more abundant in spaceflight than on earth are PHOTOSYSTEM II PROTEIN D1 (PSBA L_2_FC_Root, Shoot_=0.56, 1.02), a complex that absorbs PSII photoinhibition damages and facilitates the repair of photosystem II, and PPL1 (L_2_FC_Root, Shoot_=0.61, 1.6) a member of the oxygen-evolving complex. Likewise, ATP SYNTHASE EPSILON CHAIN (ATPBE, L_2_FC_Root, Shoot_=0.51, 0.51) was more abundant in spaceflight than on Earth in both organs (**Figure 4**). The Epsilon subunit of the F-ATPase unit selectively inhibits ATP hydrolysis. This leads to a reduced yield of ATP from photosynthesis. Of the proteins involved in the binding of ferredoxin within the reaction center, only PHOTOSYSTEM, I REACTION CENTER SUBUNIT II-1 (PSAD, L_2_FC_Root, Shoot_=0.35, 1.69) was abundant in spaceflight in both organs, while the other member of the complex were less abundant in spaceflight.

Less abundant in spaceflight in both organs are the α-subunit of CYTOCHROME B559 (PSBE, L_2_FC_Root, Shoot_=−1.37, −1.81) which reduces susceptibility to high irradiance and PHOTOSYSTEM II CP43 REACTION CENTER PROTEINs (PSBC, L_2_FC_Root, Shoot_=−0.52, −0.64, PSBB, L_2_FC_Root, Shoot_=−0.72, −1.20) which serve as the proximal antennae for Photosystem II. Arabidopsis *psbe* mutants show high susceptibility to strong light damage and reduced plastoquinone pool (Giardi et al., 2013). The decreased abundance of CYTOCHROME F (PETA, L_2_FC_Root, Shoot_=−0.77, −1.18) in the cytochrome b6/f complex in both organs suggests the exposure of the PSI to high electron influx from the PSII. The FERREDOXIN-NADP(+) OXIDOREDUCTASE-LIKE PROTEIN (PETH, L_2_FC_Root, Shoot_=−0.2, −0.29), an FNR component of the electron transport in photosystem I and the ATP SYNTHASE subunits (ATPA, L_2_FC_Root, Shoot_=−0.27, −0.24, ATPB, L_2_FC_Root, Shoot_=−0.73, −0.8, ATPC1, L_2_FC_Root, Shoot_=−0.49, −0.77) were also less abundant in spaceflight in both organs (**Figure 4**, **Supplementary Table B**). The F-type ATPase subunit drives ATP synthesis, using proton gradient, by allowing passive flux of protons across the membrane, and their reduced abundance in spaceflight coupled with increased ATP hydrolysis inhibition suggests decreased energy production via photosynthesis in spaceflight compared to earth. Notably specific to the root and more abundant in spaceflight are PSBD (L_2_FC_Root_=0.25) and PSBO1 (L_2_FC_Root_=0.5) which are mediators of oxygen evolution (Antonacci et al., 2018). The CYTOCHROME B6-F COMPLEX IRON-SULFUR SUBUNIT (PET C, L_2_FC_Shoot_=−0.64), a mediator of electron transfer from photosystem II to photosystem I was less abundant in spaceflight and specific to only the shoot (**Figure 4**). PSAD, PSBA and oxygen evolution complex proteins (PSBO, PPL1 and PSBP) had a significantly higher log2-fold increase in the shoot than in the root (**Figure 4**, **Supplementary Table B**), suggesting higher photosynthetic stress in the shoot than root despite equal exposure to light in the BRIC LED hardware.

### 3.5 Spaceflight alters ionic, molecular, and vesicular transports in both organs

**Figure 5:**
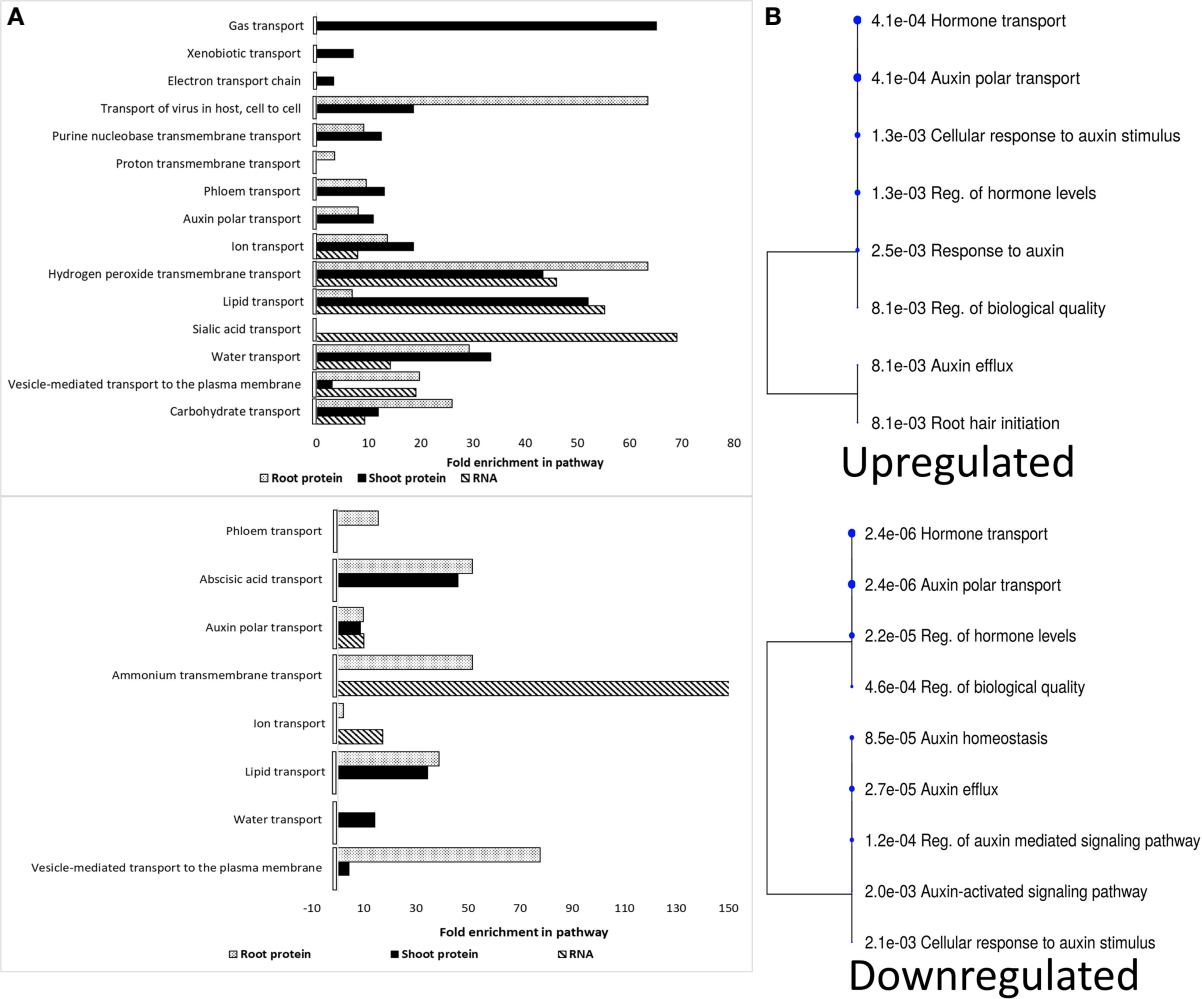
Spaceflight enrichment of RNA transcripts and proteins involved in cellular transport. **(A)**. Upregulated RNA and protein in spaceflight (top), downregulated RNA and protein in spaceflight (bottom). RNA transcripts are represented by hashed bars, shoot proteins by black bars and root proteins by dotted bars. **(B)**. Hierarchical clustering tree of enriched RNA transcripts and protein involved in auxin polar transport. The cluster tree for the upregulated auxin transporter term and downregulated auxin transporter term is bottom. Bigger dots indicate more significant *p*-values. The biological functional characterization was conducted in ShinyGO 0.77, with redundant and/or repeated GO-terms eliminated across the dataset. In the case of overlapping functional groups, the functionality with the highest enrichment is selected.

Several signaling molecules are delivered to target sites via translocation across different cellular membranes, with most ions moving passively through gated channels and molecules via active transports (Yang and Hinner, 2015). Hence, we decided to assess how the spaceflight environment affected the intracellular transport transcriptome and proteome landscape in Arabidopsis. The RNA transcript and different plant organ proteome had profoundly distinct distributions of upregulated and downregulated intracellular transporters in spaceflight (**Figure 5**). More ionic, molecular, and vesicular transport pathways were upregulated in spaceflight compared to the downregulated. Common to all the molecular fractions are RNA transcripts and proteins of auxin polar transporters. Likewise, RNA transcripts and proteins involved in the transport of reactive oxygen species (ROS), lipids, water, ions, sucrose, and vesicles were upregulated in spaceflight (**Figure 5A**).

#### 3.5.1 Ionic transport proteins

Proteins involved in ionic transport were more abundant in spaceflight across both organs (**Figure 5A**, **Supplementary Table A**). The abundance of proton and calcium-pump ATPases such as the H^+^ATPase, (AHA11, L_2_FC_Root, Shoot_=0.36, 0.99), V-TYPE PROTON PUMPS (VHAB3, VHA-A2, VHA-D, VHA-F, VHA-G2, VHA-B2 and VHA-C3) and CALCIUM-TRANSPORTING ATPASES (ACA2, ACA4, ACA8) signifies an increased effort towards maintaining cellular homeostasis in spaceflight. The calcium-transporting ATPases regulate intracellular Ca^2+^ levels and are essential for adaptation to stressful environments. The SODIUM/CALCIUM EXCHANGER (NCL, L_2_FC_Root_=0.36), also involved in the maintenance of Ca^2+^ homeostasis and the FERRITINS (FER1, FER3, FER4) were abundant in spaceflight. Ferritins provide the cell with the optimal iron level while maintaining its bioavailability for physiological conditions. The abundance of ferritin in spaceflight suggests a high requirement for iron, either as a form of defense or to protect the DNA against oxidative damage as a result of the abundance of ROS.

The organ specificity of some cation transporters is noteworthy (**Supplementary Table A** Cation transporters such as CHLORIDE CHANNEL PROTEIN CLC-A (CLC-A, L_2_FC_Root_=0.2), and POTASSIUM TRANSPORTER 13 (POT13, L_2_FC_Root_=0.37) were root-specific. Cation transporters were more abundant in the root in spaceflight than in the shoot (**Supplementary Table A**). This might be indicative of more homeostatic needs in the root than shoot in microgravity. The VACOULAR IRON TRANSPORTER PROTEIN (MEB1, L_2_FC_Root, Shoot_=1.4, −0.5) was more abundant in the root but less abundant in the shoot in spaceflight. This further confirms more iron requirements by the root in spaceflight. The CASPARIN STRIP PROTEIN (L_2_FC_Root, Shoot_=−0.62, −0.71) was also less abundant in spaceflight. CASPs are thought to direct transport across the plasma membrane by forming a protein scaffold and they also play a role in plant cell wall lignification (Kolbeck et al., 2022). Eleven of the Ras-related family proteins (RABs) were abundant in spaceflight in an organ-specific manner. These small GTP binding proteins function in targeting and tethering transport vesicles to specific acceptor compartments. The RAN GTPASE-ACTIVATING PROTEIN (RANGAF, L_2_FC_Shoot_=−0.42) was less abundant in spaceflight, while two proteins that bind to Ran GTPase homolog (RANBP1B, RANBP1C) were abundant in spaceflight than on earth (**Supplementary Table A**).

#### 3.5.2 Vesicular transport

Proteins involved in Golgi and Intra-golgi vesicular transport were more enriched on Earth than in spaceflight (**Supplementary Table A**). The majority of the VACUOLAR-SORTING RECEPTORS (VSR1, VSR2, VSR3, VSR4) with the exception of VSR7 (L_2_FC_Root, Shoot_=1.4, −0.5) were less abundant in spaceflight. The abundance of Ca^2+^ channel proteins in spaceflight is expected to lead to an abundance of VSRs as cargo binding to VSRs is enhanced by high Ca^2+^ concentration (Kang and Hwang, 2014), but this was not so. The decreased abundance of CLATHRINE HEAVY CHAIN PROTEIN (CHC2, L_2_FC_Root, Shoot_=−0.43, −0.41) in spaceflight suggests a decreased endocytosis and vesicle formation in both organs. SYNTAXIN 61 (SYP61, L_2_FC_Root_=−0.33), SYNTAXIN 81 (SYP81, L_2_FC_Shoot_=−0.57), SYNTAXIN 43 (SYP43, L_2_FC_Root_=−0.57), SYNTAXIN 22 (SYP22, L_2_FC_Root, Shoot_=0.46, 1.48) and SYNTAXIN 132 (SYP132, L_2_FC_Root_= 0.34), member of SNARE proteins, were organ-specific in their abundance in spaceflight. The NOVEL PLANT SNARE 11 (NPSN11, L_2_FC_Root, Shoot_=1.27, 1.14) was also abundant in both organs in space (**Supplementary Table A**). Synaptotagmins (SYTA L_2_FC_Root_=0.29), a calcium sensor that regulates rapid and synchronous vesicle exocytosis for plant viral movement and a requirement for tethering the ER to the plasma membrane, was also abundant in space.

#### 3.5.3 Lipid and hormone transport

RNA transcripts and proteins involved in the intracellular transportation of lipids were more enriched and abundant in spaceflight than on Earth (**Figure 5**). GLYCOLIPID TRANSFER PROTEIN (GTP, L_2_FC_Root_=0.32) and ACCELERATED CELL DEATH 11 (ACD11, L_2_FC_Shoot_=0.32), involved in sphingolipid transport were abundant in spaceflight in both root and shoot respectively. The abundance of NON-SPECIFIC LIPID-TRANSFER PROTEINS (LTP1, LTP5, LTPG31) and PROTEIN FATTY ACID EXPORT 3 (FAX3, L_2_FC_Root, Shoot_=0.32, 0.66) in spaceflight suggests the use of long-distance systemic signaling in space and the formation of lipid polymer barriers. The PHOSPHOLIPID-TRANSPORTING ATPASE 10 (ALA10, L_2_FC_Root, Shoot_=0.69, 0.55), a lipid flippase, was abundant in spaceflight. ALA10 is involved in the desaturation process of phosphatidylcholine, a lipid abundant during phosphate starvation and in the regulation of auxin polar transport. The abundance of PATELLINs (PATL2, PATL4, PATL6), regulators of auxin-mediated PIN1 relocation, and AUXIN EFFLUX CARRIERS 3 (PIN3, L_2_FC_Root_=0.49) in spaceflight and the decrease in abundance of the PIN4 (_L2FC Root/Shoot_=−0.46, −0.75), PIN7(_L2FC Root/Shoot_=−0.27, −0.84), AUXIN RESPONSE 4 (AXR4, L_2_FC_Root, Shoot_=−0.34, −0.47), AUXIN TRANSPORTER PROTEIN1 (LAX1, L_2_FC_Shoot_=−0.74), ABCB1 (L_2_FC_Shoot_=−0.21) and ABCB20 (L_2_FC_Root, Shoot_=−0.35, −0.65) suggests an alteration in polar auxin redistribution in plants grown in spaceflight (**Figure 5B**). The MYOSINs (XIB, ATM1, XI-H, XI-K, MYA2), which are motor proteins that drive movement along the actin filaments were not significantly abundant (*p*<0.05) in the shoot in spaceflight but were significantly less abundant in the root. The expression of auxin transporters was organ-specific and this suggested that the distribution of auxin is altered differently in the root and shoot for adaptation to the space environment (**Supplementary Table A**).

### 3.6 Influence of spaceflight on proteins involved in cell wall synthesis, integrity and maintenance

Altered gravity has been identified to have profound effects on the architecture of plant cell walls (Kordyum and Chapman, 2017). Common to both organs and more abundant in spaceflight than on Earth are Endoglucanase 20 (L_2_FC_Root, Shoot_=0.86, 1.13), involved in the degradation of cellulose to its monomeric subunit, FASCICLIN-LIKE ARABINOGALACTAN PROTEINs (FLA 7, L_2_FC_Root, Shoot_=1.51, 1.43, and FLA 9, L_2_FC_Root, Shoot_=0.73, 0.53) and PLASMA MEMBRANE-ASSOCIATED CATION-BINDING PROTEINs (PCAP1, L_2_FC_Root, Shoot_=0.49, 0.52 and PCAP2, L_2_FC_Root, Shoot_=1.08, 1.47) which are have been implicated in microtubule destabilization and identified as possible regulators of phosphatidylinositol phosphates membrane reformations during intracellular signaling (**Table 2**). VILLINs (VLN2, VLN3, VLN4), proteins involved in the bundling, severing, nucleating, and capping of the actin cytoskeleton, were also more abundant in spaceflight than on Earth, in both organs (**Table 2**). Spaceflight alters the biogenesis of microtubules and this is achieved differently in the root and shoot.

**Table 2:** Differentially abundant proteins related to cell wall biosynthesis and maintenance.

TAIR.ID	Protein ID	Symbol	Root	Shoot
			L_2_FC	L_2_FC
AT4G23560	Endoglucanase 20		0.86	1.13
AT5G49360	Beta-D-xylosidase 1	BXL1	0.48	–
AT4G24510	Protein ECERIFERUM 2	CER2	0.2	0.68
AT4G32410	Cellulose synthase A catalytic subunit 1 [UDP-forming]	CESA1	−0.44	–
AT5G05170	Cellulose synthase A catalytic subunit 3 [UDP-forming]	CESA3	−0.44	–
AT5G09870	Cellulose synthase A catalytic subunit 5 [UDP-forming]	CESA5	−0.52	–
AT5G64740	Cellulose synthase A catalytic subunit 6 [UDP-forming]	CESA6	−0.51	−0.83
AT5G17420	Cellulose synthase A catalytic subunit 7 [UDP-forming]	CESA7	−2.37	–
AT1G55850	Cellulose synthase-like protein E1	CSLE1	−0.22	−0.33
AT4G23990	Cellulose synthase-like protein G3	CSLG3	−0.51	–
AT2G04780	Fasciclin-like arabinogalactan protein 7	FLA7	1.51	1.43
AT1G03870	Fasciclin-like arabinogalactan protein 9	FLA9	0.73	0.57
AT2G36250	Tubulin/FtsZ family protein	FTSZ2-1	−0.36	−0.42
AT5G06680	Gamma-tubulin complex component 3	GCP3	−0.25	−0.45
AT3G01640	Glucuronokinase 1	GLCAK1	0.28	–
AT4G20260	Plasma membrane-associated cation-binding protein 1	PCAP1	0.49	0.52
AT5G44610	Plasma membrane-associated cation-binding protein 2	PCAP2	1.08	1.47
AT3G02230	UDP-arabinopyranose mutase 1	RGP1	0.22	–
AT5G15650	UDP-arabinopyranose mutase 2	RGP2	0.56	–
AT3G05530	26S proteasome regulatory subunit 6A homolog A	RPT5A	0.35	0.77
AT3G16640	Translationally controlled tumor protein 1	TCTP1	0.38	–
AT3G10220	Tubulin-folding cofactor B	TFCB	−0.4	–
AT1G64740	Tubulin alpha-1 chain	TUBA1	–	−0.78
AT5G19770	Tubulin alpha-5 chain	TUBA5	–	−0.3
AT5G62690	Tubulin beta-2 chain	TUBB2	0.23	–
AT5G44340	Tubulin beta-4 chain	TUBB4	0.36	–
AT2G29550	Tubulin beta-7 chain	TUBB7	−0.41	–
AT5G23860	Tubulin beta-8 chain	TUBB8	0.43	–
AT4G20890	Tubulin beta-9 chain	TUBB9	0.6	–
AT5G05620	Tubulin gamma-2 chain	TUBG2	−0.31	–
AT1G76030	V-type proton ATPase subunit B1	VHA-B1	0.16	0.23
AT2G41740	Villin-2	VLN2	0.39	0.6
AT3G57410	Villin-3	VLN3	0.34	1.12
AT4G30160	Villin-4	VLN4	0.39	0.49

The majority of the differentially abundant cell wall-related proteins between spaceflight and Earth were specific to the root. CELLULOSE SYNTHASES (CESA1, CESA3, CESA5, CESA7) and CELLULOSE-LIKE PROTEINS (CSLE1, CSLG3), involved in cellulose deposition and TUBULIN COFACTOR B (TFCB, L_2_FC_Root_=−0.4), whose expression reduces the number of microtubules, were root-specific and less in spaceflight (**Table 2**). β-tubulin dimers (TUBB2, TUBB4, TUBB8, TUBB9) were abundant in spaceflight in the root alone. β-tubulins are more exposed to phosphorylation than α-tubulin which are prone to detyrosination. Modification of tubulin is usually mediated by the tubulin cofactors. The UDP-ARABINOPYRANOSE MUTASEs (RGP1, L_2_FC_Root_=0.22 and RGP2, L_2_FC_Root_=0.56) involved in the biosynthesis of cell wall non-cellulosic polysaccharides were abundant in the root in spaceflight. Specific to the shoot are the polymers of α-tubulin, TUBA1 (L_2_FC_Shoot_=−0.78) and TUBA5 (L_2_FC_Shoot_=−0.3), which were less abundant in spaceflight compared to Earth (**Table 2**).

### 3.7 Spaceflight and the plant REDOX homeostasis

**Figure 6:**
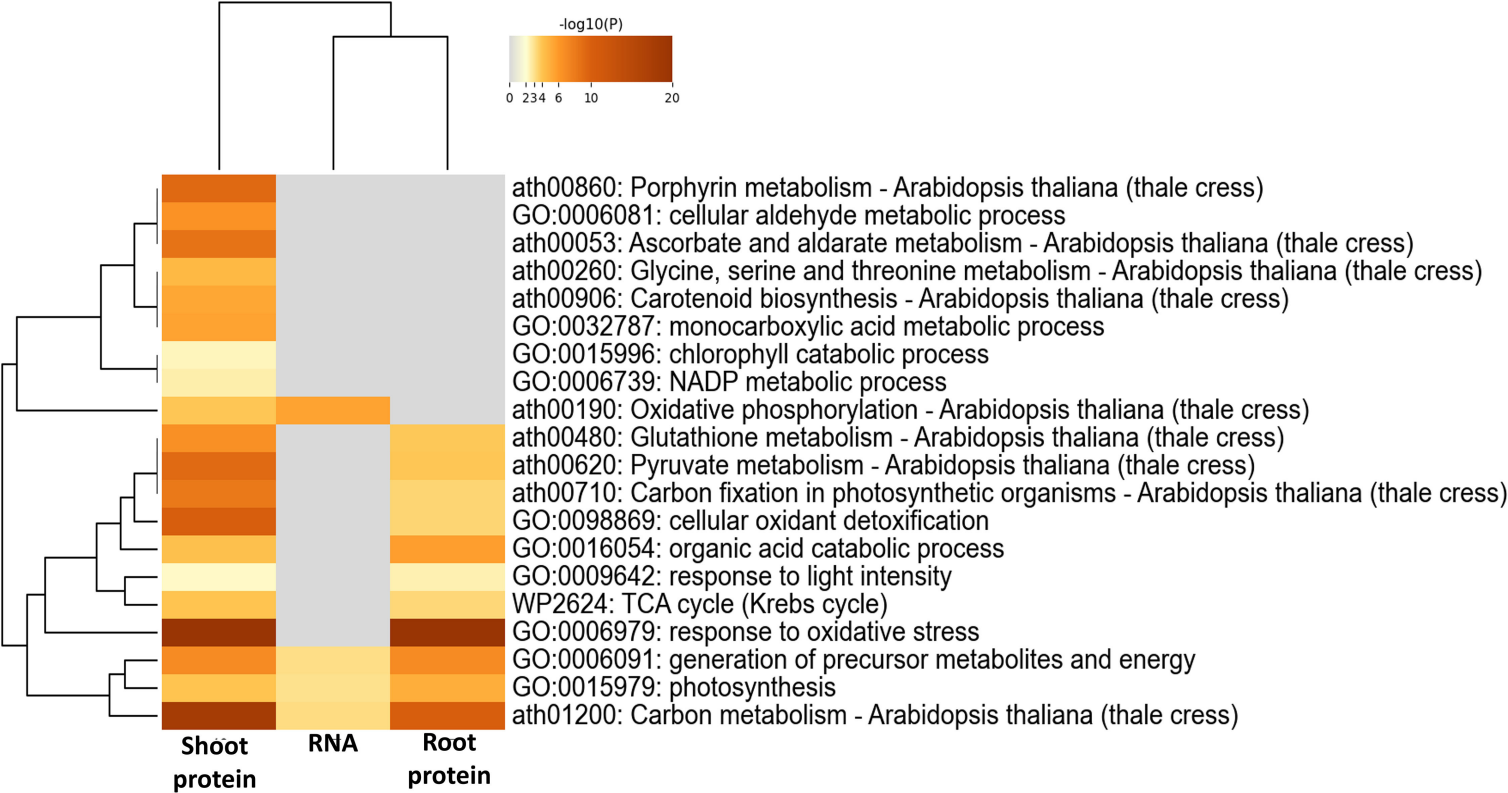
Redox homeostasis in spaceflight. Heatmap of GO enrichment terms (molecular and biological) of RNA transcripts, shoot proteins, and root proteins involved in the cellular response to oxidative stress and maintenance of redox homeostasis. The color gradient represents the significance of the enrichment of the genes and proteins in the metabolic pathway indicated by the right side of the plot.

Regulating the accumulation of reactive oxygen species (ROS), antioxidants and redox enzymes in plant cells is a key adaptation mechanism for plant survival. ROS are involved in plant systemic signaling, and at the same time can be a result of stress metabolism. Over 132 RNA transcripts and 241 proteins involved in the maintenance of cellular redox homeostasis and ROS signaling were significantly altered (*p*<0.05) in spaceflight (**Figure 6**, **Supplementary Table C**). While the RNA transcripts included only a few of the traditionally known ROS and ROS-associated genes such as peroxidases, -redoxins, dehydrogenases, and so on, the proteome had many of them. The RNA transcripts involved in the response to ROS pathway differentially expressed in spaceflight include heat shock proteins (HSPs), transcription factors, and ubiquitin ligases (**Figure 6**). In both organs, 18 of the 20 PEROXIDASE proteins were abundant in spaceflight, indicating a high rate of ROS generation through the reduction of peroxide. Proteins regulating isocitrate metabolism, AC01 (L_2_FC_Root_=0.33), ACO3 (L_2_FC_Root_=0.57), CICDH (L_2_FC_Root_=0.54), and IDH5 (L_2_FC_Shoot_=0.43) were more abundant in spaceflight than on earth. The isocitrate pathway is the branchpoint of many other metabolic pathways in plants and the abundance of the Isocitrate dehydrogenase NAD+ catalytic enzymes suggests that plants in spaceflight environments use the isocitrate pathway to supplement NADPH generation from the pentose phosphate pathway.

SUPEROXIDE DISMUTASE, FSD1 (L_2_FC_Root, Shoot_=0.9, 0.99), FSD3 (L_2_FC _Shoot_=0.31) and THIOREDOXIN REDUCTASE (NTRA, L_2_FC_Root, Shoot_=1.0, 193) were abundant in spaceflight and they facilitate the removal of superoxide in an NADPH-dependent manner. A vacuolar phosphatase (PAP26, L_2_FC_Root, Shoot_=0.43, 0.76) involved in the recycling of inorganic phosphate from intracellular phosphate metabolite (Veljanovski et al., 2006) was more abundant in spaceflight than on Earth. Enriched in the spaceflight environment are proteins (PGD1, TKL-2, NADP-ME2, and G6PD1), recognized as major participants of the pentose-phosphate shunt pathway (**Supplementary Table C**). The pentose phosphate shunt pathway is a parallel alternative to the glycolysis pathway. Surprisingly, both RESPIRATORY BURST OXIDASE HOMOLOG PROTEIN D (RBOHD, L_2_FC_Shoot_=−0.67) and L-ASCORBATE PEROXIDASE 3, L_2_FC_Shoot_=−0.95) were less abundant in spaceflight in the shoot. The RBOHD produces ROS in response to environmental stress and plant signaling. Here, roots seem to be more impacted by oxidative stress and abundance of ROS than shoots in spaceflight (**Supplementary Table C**).

## 4 Discussion

### 4.1 Arabidopsis adapts photosystems to the spaceflight environment to minimize impacts on photosynthesis

This study assesses the physiological and molecular impacts of the spaceflight environment on Arabidopsis seedlings grown in NASA BRIC LED hardware using a combined transcriptomics and proteomics approach. The BRIC LED hardware, with integrated LED illumination, provides the growing seedlings with a source of light. Hence, plants growing in the BRIC LED hardware on ISS will have light cues but lack gravitational cues. This enabled us to study the effects of microgravity on plant transcriptome and organ-specific proteome, while also providing insight into how the plant root and shoot individually integrate light signaling in the absence of gravity. Evidence from this study suggests that the operations of the photosynthetic machinery (PSII and PSI) were greatly impacted in spaceflight (**Figure 4**), with the plant shifting photosystem core-regulatory proteins in an organ-specific manner to adapt to the microgravity environment. While the net photosynthetic rate might not be affected in microgravity as it was not captured in this study, the molecular functions of the photosynthetic apparatus, as revealed by this study, were impacted. The abundance of the NusG-like proteins (PTAC12, PTAC14, PTAC3, PTAC4) in the shoot and NusG-like proteins (PTAC16, PTAC14) in the root (**Supplementary Table D**) suggests attempts at boosting the translational efficiency of chloroplast ribosomes to reduce the plant sensitivity to environmental stress in spaceflight. The abundance of D1 complex protein (PSBA) and the PPL1 in spaceflight suggests attempts to optimize the water-oxidizing reaction and repair damages to the PSII. Reduced abundance of the PSAB and PSAE isoforms of the PSI in spaceflight, signifies an increased susceptibility of the PSII to photoinhibition (Krieger-Liszkay et al., 2020; Ji et al., 2022) and a deficiency of the PSI steady state redox-level and re-oxidation. The PPL1, which was abundant in spaceflight in both organs, works to facilitate the assembly of PSII and optimize plant fitness under increased sensitivity to the BRIC LED hardware light.

Suppression of the PSI electron transport subunit protein, PETH, in spaceflight, is associated with reduced efficiency of the electron transport chain and instability of the PSI. However, this reduced efficiency is compensated for by an increased abundance of chloroplast gene translations (PTACs), which helps to prevent over-reduction of the plastoquinone pool. The reduced abundance of PSII proximal antennae protein cp43 (PSBC) and the light-harvesting chlorophyll complexes (LHCB1, LHCB4, LHCB5, LHCB6) in spaceflight suggest an attempt to decrease light harvest by the photosystems, hence decrease photoinhibition, increase the oxygen-evolving capability and improve repair to PSII. Varying degrees of molecular perturbations to the plant photosystem complexes in spaceflight have earlier been reported, which further validate our findings (Stutte et al., 2005; Chen and Wang, 2016; Vandenbrink et al., 2019). However, unlike in this current study, where PSII and PSI complexes transcripts (RNA) were upregulated in spaceflight, Vandenbrink et al. (2019) reported significant downregulation of transcripts enriched in photosynthetic pathways in spaceflight compared to ground samples. They also indicated the downregulation of four RNA transcripts associated with photosynthetic electron transport (petE: plastocyanin, petF: ferredoxin, petH: ferredoxin–NADP+ reductase, petJ: cytochrome c6). These transcripts were however upregulated in spaceflight in our current study, but they were all downregulated at the proteome level in both the plant root and shoot (**Figure 4**). These discrepancies at the transcript level may be due to the difference in Arabidopsis wildtype ecotypes and spaceflight hardware utilized in both experiments. While Vandenbrink et al. (2019) utilized the *Arabidopsis thaliana* ecotype Landsberg and the European Modular Cultivation System (EMCS) hardware, this study utilized the *Arabidopsis thaliana* ecotype Columbia-0 and the BRIC LED hardware. Basu et al. (2017) and Olanrewaju et al. (2023) emphasize that the choice of hardware is a significant source of variations in the results of plant spaceflight experiments transcriptomics and proteomics.

Chen and Wang (2016) delineated another tangent by revealing that out of 38 photosystem-associated proteins that exhibited differential expression in spaceflight-grown rice seedlings, 34 were downregulated in spaceflight compared to Earth controls. While our findings and that of Vandenbrink et al. (2019) support the coherent perturbation of both the PSII and PS I as revealed by the transcriptome and proteome, Chen and Wang (2016) suggested from their fluorescent emission spectra studies that despite the changes to the PSII proteins in spaceflight, their activity under microgravity is not affected, in contrast with the PSI which had significantly reduced energy transfer rates, hence impaired photosynthetic activity. Of course, Chen and Wang (2016) study was carried out on Rice seedlings, not Arabidopsis and directly extrapolating the differential PS I and PSII findings to Arabidopsis might not be so efficient. However, a consensus is that the spaceflight environment leads to a reduction in the PS I and PSII complexes, which may be accompanied by reduced photosynthetic efficiency and consequent smaller plant tissues.

The expression of the photosynthesis-associated proteins in the root might be due to the architecture of the BRIC LED hardware, which places the LEDs in close proximity to the roots, making them prone to direct hit by light. Interestingly, Paul et al. (2013) also reported the expression of several genes involved in light regulation in Arabidopsis roots in spaceflight transcriptomics experiments, even when the roots were not in close proximity to light. This phenomenon was also observed by Kruse et al. (2020) in their whole seedling transcriptomics study, despite that the seedlings were grown in the total absence of light cue in spaceflight. Hence, it is suggested that when gravity is absent, plant roots rely more heavily on negative phototropism for navigation, inducing light-sensing “tools” in roots in the spaceflight environment (Paul et al., 2017). The proteome landscape of the root and shoot, however, differs significantly in spaceflight, suggesting different adaptation mechanisms to the space environment, a further confirmation of Paul et al. (2013) transcriptomic study’s observation of organ-specificity in plant response to spaceflight. Vandenbrink et al. (2016) also proposed an interaction between plants’ gravity and light harvesting machinery at a genetic level, based on their observations of blue-light phototropic response in roots of Arabidopsis grown in microgravity.

The upregulation of most of the photosystem and contrasting decreased abundance of corresponding proteins in spaceflight (**Figure 4**) however, suggests that major adaptive response to spaceflight environmental stimulus might be regulated at the post-transcriptional level, even though the direct effect of the stimulus is still observed at the transcriptional level. A combined translatome-proteome study approach will be useful to elucidate this phenomenon.

### 4.2 Intracellular trafficking and cell wall synthesis in spaceflight

Plants, throughout their evolutionary history, have developed remarkable adaptations to counteract the unrelenting force of gravity (Medina et al., 2021). To withstand the gravitational force, plants have to ensure a balance between the internal turgor pressure, mediated by intracellular trafficking, and tensile resistance of both the primary and secondary cell walls. One pivotal molecular insight is the potential shift in cellulose metabolism in spaceflight, as indicated by the heightened abundance of Endoglucanase 20. Enhanced cellulose degradation might be a strategic adaptation, possibly optimizing cell wall flexibility in response to the unique stresses of a microgravity environment. This could be complemented by shifts in cellular adhesion dynamics, given the altered levels of FASCICLIN-LIKE ARABINOGALACTAN PROTEINs. The implications of these shifts could range from altered tissue integrity to modifications in intercellular communication. In a comprehensive transcriptomics meta-analysis of Arabidopsis plants cultivated in spaceflight within the BRIC Petri-dish fixation unit (PDFU) hardware, Johnson et al. (2017) highlighted potential alterations in the cell wall matrix of plants in spaceflight, as revealed by the glycomics evaluation of the BRIC-16-Cyt samples. They implied that microgravity influences the biosynthesis of xylan and pectic elements, leading to variations in the composition of the cell wall matrix.

Alongside this, Johnson et al. (2017) also reported the reduced abundance of xylan and pectic epitopes in spaceflight-grown plants, which resonate with our findings, highlighting the potential changes in the biosynthesis of cell wall non-cellulosic polysaccharides in spaceflight conditions, particularly in the root. Nakashima et al. (2023) in their novel glycome profiling and immunohistochemistry of Arabidopsis in spaceflight also observed that roots from spaceflight Arabidopsis seedlings displayed a generally increased labeling intensity when targeted with xyloglucan monoclonal antibodies (mAbs) compared to ground controls. They concluded that the heightened fluorescence intensity post-labeling with xyloglucan mAbs might result from changes in polysaccharide biosynthesis and secretion due to microgravity conditions. Wakabayashi et al. (2020) in their studies on Rice seedling cell wall under spaceflight conditions indicated that microgravity reduces the 1,3:1,4-β-glucan content in rice shoot cell walls, implicating an increase in the expression of an endo-1,3:1,4-β-glucanase gene. This reduction might serve to keep the cell wall more flexible under microgravity conditions. This observation is in harmony with our data, revealing potential changes in the biosynthesis of cell wall non-cellulosic polysaccharides, in spaceflight environments. Interestingly, while these studies reported that glucan metabolism is impacted, the spaceflight environment (microgravity) seems to exert minimal influence on the metabolism of arabinoxylans. This differentiation in response indicates a level of specificity in how plants adjust their cell wall components in response to altered gravity.

In the absence of gravity, seedlings have to default to secondary signals such as other environmental impulses. This affects internal homeostasis, inter- and intra-cellular transports, and cell wall composition. Our results identified intracellular Ca^2+^ transport as a major player in adaptation to microgravity in both root and shoot. This is characterized by the increased abundance of the vacuolar proton ATPases and calcium-transporting ATPases. The vacuolar ATPases, contributing to the cytosolic pH homeostasis, form electrochemical proton gradients and drive active transport into cell organelles and they are important targets of phosphorylation and redox modification. The root-specific abundance of the cation transporters suggests that the homeostatic needs of the root differ from that of the shoot in spaceflight. The altered abundance of small GTPases such as Ran, Rab, and Ras, between spaceflight and Earth suggests a functional dysregulation of vesicle trafficking in spaceflight. The spaceflight environment alters molecular switches between the membrane-bound active GTP and cytosolic inactive GDP which tremendously affects signal transduction.

Molecular mechanisms driving polar auxin distribution were affected and organ-specific, with the altered abundance of auxin transporters in spaceflight and on Earth. However, auxin transport and response appear to be more pronounced on Earth than in microgravity. Results from this study also suggest a decreased stability of the cellulose cell wall in spaceflight, due to the abundance of cellulose-degrading proteins and less abundance of cellulose synthase (**Table 2**). The spaceflight environment led to the decreased formation and increased destabilization of the microtubules which suggests that seedlings seem to fall back on the actin skeleton for support. Interactions between PIP2 which is abundant in space, with VILLINs which were also abundant in space inhibit the capping and severing actions of actin but rather increase actin binding at the headpiece region. This suggests that actin cross-nucleation and cross-linking is an adaptation mechanism for plants in space. An in-depth study into vesicle and cargo sorting under microgravity will give more insight into how plants adapt their intracellular trafficking and cell wall depositions to the spaceflight environment.

### 4.3 Spaceflight significantly affects plant translation machinery in an organ-specific pattern

The varied abundance of over 150 ribosomal subunit proteins, spliceosomes and proteasomes in spaceflight and across both organs suggest major alterations to plant transcriptional and translation machinery as an adaptation mechanism to the spaceflight environment (**Supplementary Tables E, F**). The decreased abundance of various mRNA slicing factors (ATO, CUV, SC35, SCL30A, U2AF65A, U2AF65B, DEAH3) in the shoot in spaceflight suggests less mRNA splicing in the shoot compared to the root, which might lead to the abundance of non-functional proteins in the shoot and increased need for proteasome activities. Combining transcript data with this proteome data will give more insight into this. The abundance of HEAT SHOCK PROTEIN families (HS60, HSP70, HSP90) in both organs suggests an increased control of protein homeostasis in space characterized by folding of nascent polypeptides released from the ribosome, sorting of proteins to cell organelles and providing a link to the ubiquitin-mediated proteasome degradation. The relative abundance of 26S proteasomes involved in the degradation of misfolded and orphaned proteins suggests that the spaceflight environment led to errors in protein translation, hence the need to increase the efficiency of the proteasome unit.

### 4.4 Combating oxidative stress while maintaining ROS signaling in spaceflight

Seedlings in spaceflight are faced with the need to maintain a balance between the abundance of ROS for systemic stress signaling and their damaging effects. Our results suggest dedicated efforts to the removal of superoxide radicals by the abundance of several peroxides, thioredoxin, ferritin, and superoxide dismutase in spaceflight. We infer that plants in spaceflight utilize a shunt pathway, which is a parallel alternative to the glycolytic pathway, to complement the NADPH production from the pentose-phosphate pathway. The pentose-phosphate shunt pathway is driven by NADPH demands in different tissues (Aziz and Mohiuddin, 2022), and in this extremity, plants in spaceflight recycle inorganic phosphate from intracellular phosphate metabolites in the vacuole, mediated by PAP26 to meet the NADPH demands. This suggests that the metabolic energy requirement for plants in space is higher than on Earth. The abundance of aldehyde dehydrogenases (ALDHs) and several glutathione dehydrogenases suggests the involvement of the S-nitrosation reaction in cellular homeostasis in spaceflight. A common feature of the ALDHs is the susceptibility of their catalytic cysteine residue to GSNO-mediated S-nitrosation (Stiti et al., 2020), which has been suggested as a central gravitropism signaling event (Kruse and Wyatt, 2022).

While this study substantiates that the BRIC LED hardware supports Arabidopsis plant growth in spaceflight, it is important to note that the BRIC hardware, unlike some newly developed advanced spaceflight hardware such as the Advance Plant Habitat (APH), is a closed system, hence no gas exchange between the plant and the external environment. This is a major limitation for the hardware, as this promotes the buildup of gases such as ethylene, oxygen and other volatile organic compounds (VOCs) (Klymchuk et al., 2003). Despite being an important hormone to plant, elevated ethylene level had been identified to cause anomalous Arabidopsis plant growth in spaceflight (Stankovic, 2018) and sterility in male wheat plants grown in spaceflight (Campbell et al., 2001). Beyond ethylene, the accumulation of oxygen and other VOCs can potentially reach toxic levels in the BRIC LED hardware. This is a substantial source of stress as indicated by Basu et al. (2017). Drawing parallels with Earth, closed environments like terrariums might encounter similar challenges with gas accumulation. However, there’s a critical difference. On Earth, natural processes and microbial interactions often intervene to balance out the system over time. But in the unique microgravity setting of space, these natural balancing acts might not function as they do here, thereby amplifying the challenges of a closed system, as we consider plants for bio-regenerative life support in long-term spaceflight missions.

### 4.5 Non-correlated transcriptome and proteome: indicative of post-transcriptional regulation in spaceflight?

Spaceflight environmental conditions pose unique challenges to plants, leading to significant alterations in gene expression and protein synthesis. Our analysis of Arabidopsis plants grown in space reveals that the expression of RNA transcripts did not correspond to their translation to shoot and root proteins. The observed non-correlation between transcriptome and proteome data from Arabidopsis plants grown in spaceflight may indeed suggest a prevalent post-transcriptional regulation (Wang et al., 2022). Transcript-protein discordance can be attributed to multiple factors. One such factor is the differential extraction protocols and data processing for RNA and protein, which can introduce variation in the extracted quantities (Liu et al., 2016). Furthermore, due to the unique environmental stressors of spaceflight, the mechanisms to arrest translation could be compromised, resulting in skewed protein synthesis rates. Moreover, the phenomenon of alternative splicing, often upregulated under stress conditions, can result in multiple protein isoforms from a single transcript, further complicating the correlation. Another source of discordance to note is that the RNA transcriptome might be a reflection of the plant’s immediate response to stress, while the proteome represents a more stabilized, adaptive response. These unique constraints of spaceflight can thus evoke distinct reactions at plants’ transcriptome and proteome levels, leading to non-correlated outcomes. Hence, attributing the non-correlation of the transcriptome and proteome only to alternative splicing will be a bias.

This study, by combining transcriptomics and proteomics, provided insight into plants’ adaptive responses to the spaceflight environment at multiple molecular levels. While we did not directly measure photosynthetic net production, our findings indicate a series of modifications to photosystems II and I aimed at adapting to the space environment, potentially decreasing the plant’s sensitivity to light intensity and enhancing its oxygen-evolving capacity. Concurrently, we observed evidence for an increased metabolic energy requirement in spaceflight, with plants seemingly engaging alternate energy generation pathways to complement photosynthetic production. Furthermore, our findings indicate significant impacts on cellular trafficking and potential compromises to cell wall integrity, suggesting an adaptation in structural support provided by actin filament cross-linking and cross-nucleation. This research not only serves as a comprehensive study of plant response in spaceflight but also holds profound implications for terrestrial agriculture, providing valuable insights for developing crop varieties resilient to environmental stressors on Earth, such as drought, extreme temperatures, and salinity.

This study also contributes to the broader goal of space agriculture – a key component of long-duration space missions and off-Earth colonization. While centered around the model plant Arabidopsis, our findings could provide the foundation for developing crop varieties suitable for growth in extraterrestrial environments, supporting food production for future missions to Mars and the Moon. Thus, the findings of our research, while intrinsically fascinating, also bear practical implications for the future of both terrestrial and space agriculture. As we continue to explore the final frontier, understanding how life – in this case, plant life – adapts and thrives is crucial for the success of our extraterrestrial endeavors and the sustainable development of our agriculture on Earth.

## Data availability statement

The datasets presented in this study can be found in online repositories. The names of the repository/repositories and accession number(s) can be found below: https://osdr.nasa.gov/bio/repo/data/studies/OSD-522/preview/w5Q5ElZE-Wy6DDS1ZFzeRjBAPuKss-x0, OSD-522.

## Author contributions

GO: Conceptualization, Data curation, Formal Analysis, Investigation, Methodology, Project administration, Resources, Software, Validation, Visualization, Writing – original draft, Writing – review & editing. NH: Conceptualization, Formal Analysis, Investigation, Software, Writing – original draft, Writing – review & editing. MN: Formal Analysis, Investigation, Software, Writing – review & editing. AP: Conceptualization, Funding acquisition, Project administration, Resources, Supervision, Validation, Writing – review & editing. RF: Conceptualization, Funding acquisition, Project administration, Resources, Supervision, Validation, Writing – review & editing. SW: Conceptualization, Formal Analysis, Funding acquisition, Investigation, Project administration, Resources, Supervision, Visualization, Writing – review & editing.
