# Supplementary material for: Integrative transcriptomics and proteomics profiling of Arabidopsis thaliana elucidates novel mechanisms underlying spaceflight adaptation
Source: Front Plant Sci. 2023 Nov 27;14:1260429. doi: 10.3389/fpls.2023.1260429 (PMC10712242; doi:10.3389/fpls.2023.1260429)
Supplement: Supplementary file 1 [file DataSheet_1.pdf]

SUPPLEMENTARY FIGURES

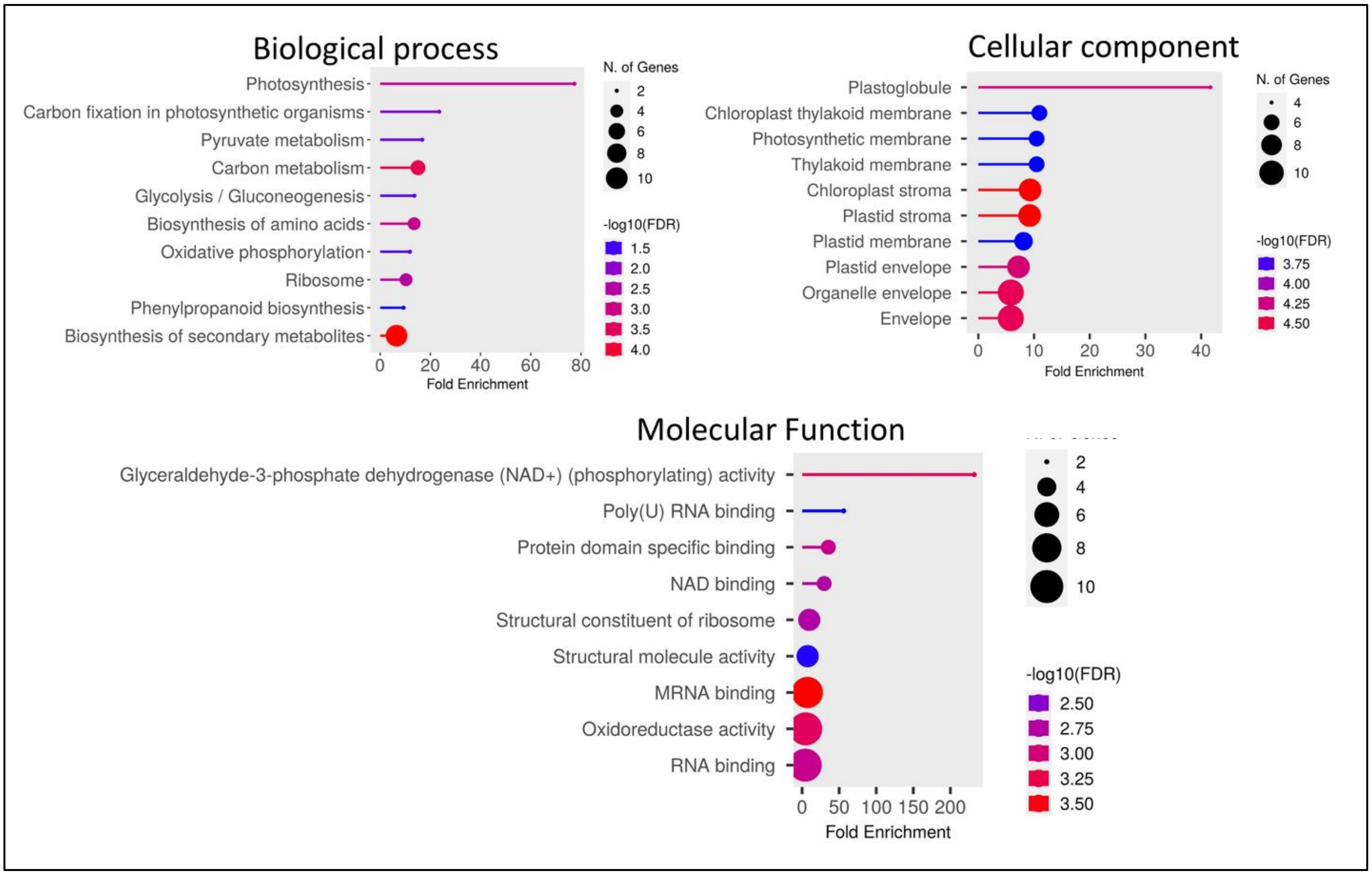

Supplementary Figure 1: A KEGG pathway analysis of the 34 membrane proteins with increased abundance in the shoot and decreased abundance in the root in spaceflight.

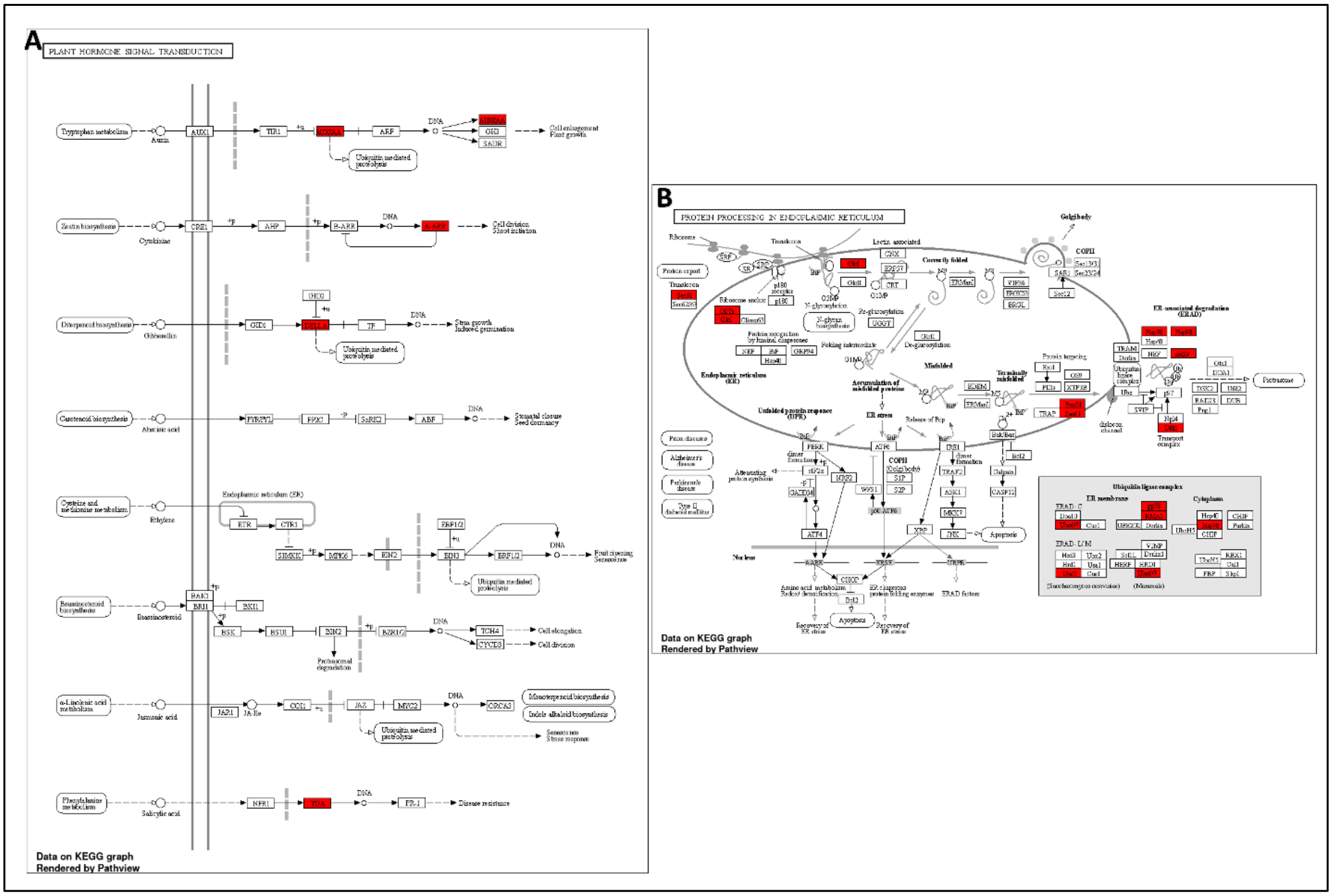

Supplementary Figure 2: KEGG pathway analysis. **A.** KEGG pathway view of the nucleus-targeted transcripts upregulated in spaceflight indicated. **B.** KEGG pathway view of the nucleus-targeted transcripts downregulated in spaceflight indicated.

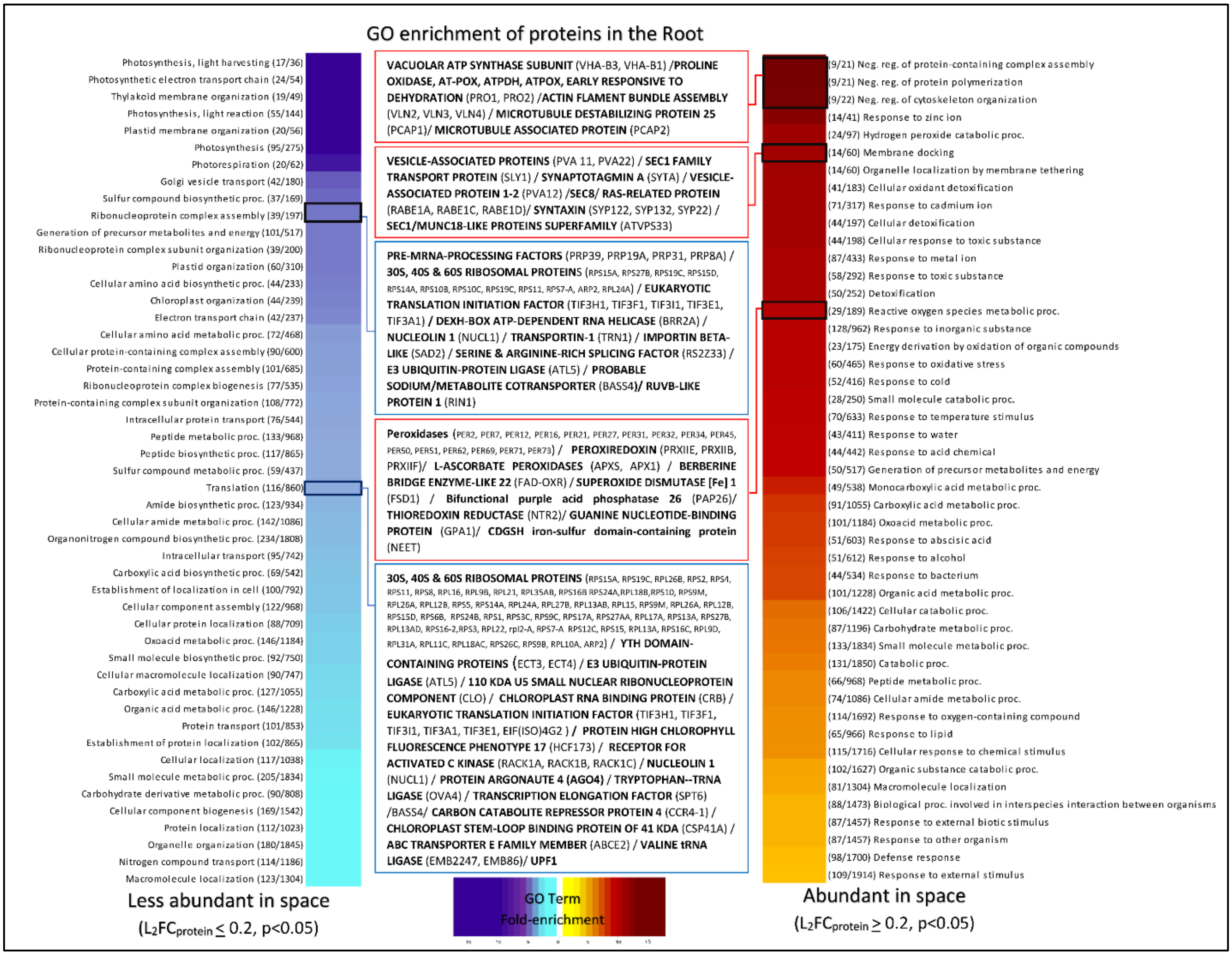

Supplementary Figure 3: GO enrichment of differentially abundant proteins expressed in the root in spaceflight and on Earth.

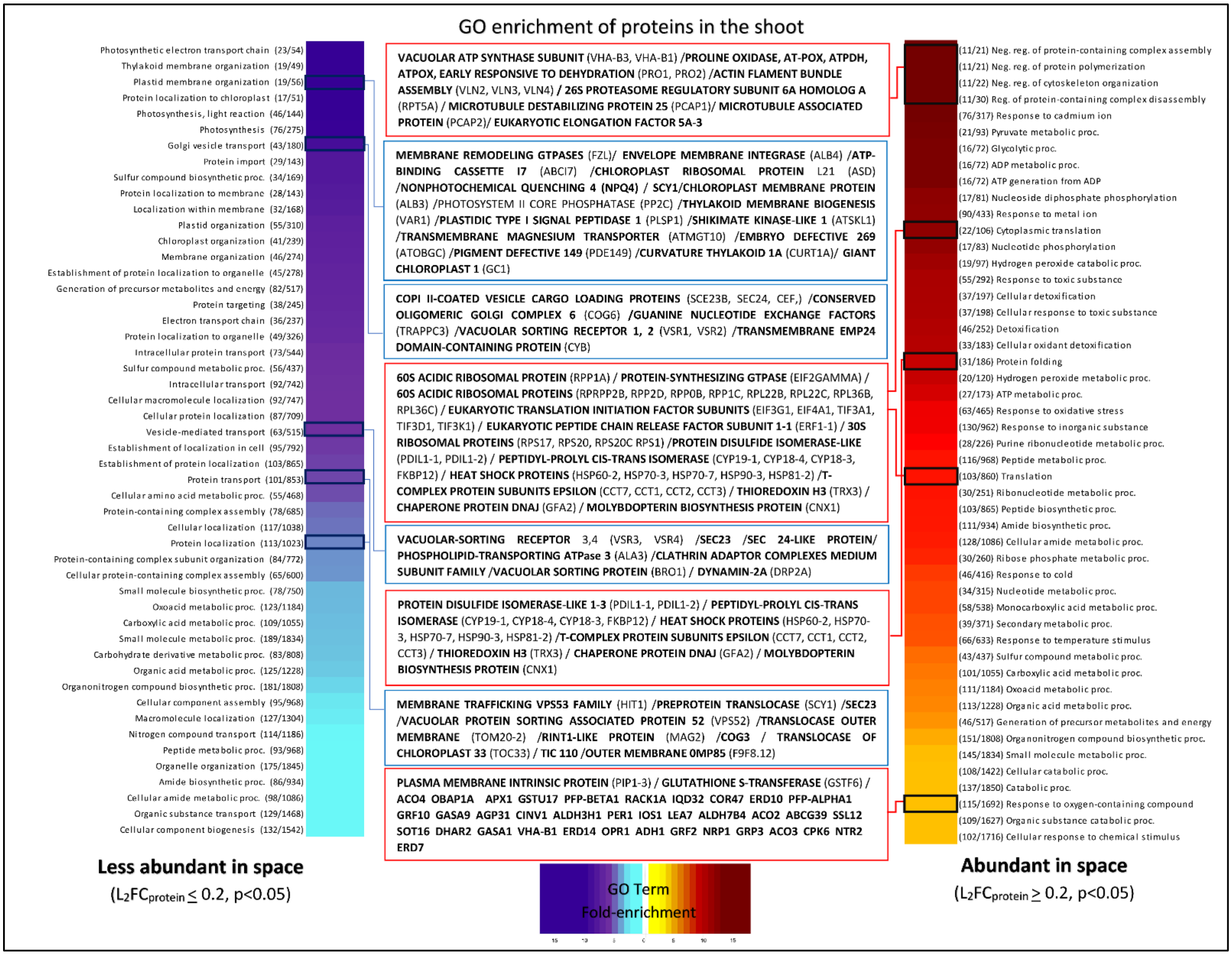

Supplementary Figure 4: GO enrichment of differentially abundant proteins expressed in the shoot in spaceflight and on Earth.

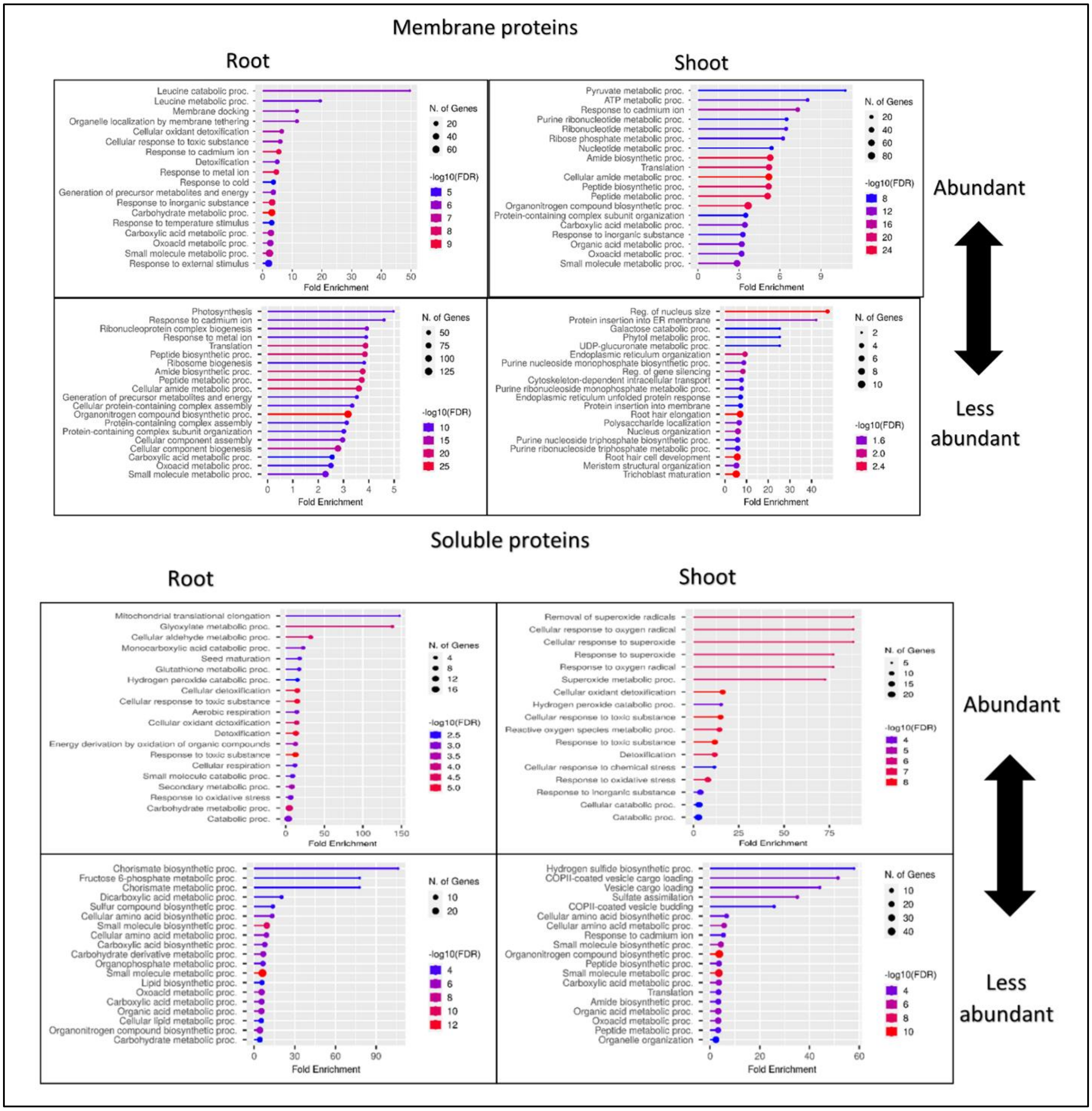

Supplementary Figure 5: Enrichment analysis of the soluble and membrane proteins that are differentially expressed in root and shoot in spaceflight and on Earth.
