# Supplementary material for: Integrative transcriptomics and proteomics profiling of Arabidopsis thaliana elucidates novel mechanisms underlying spaceflight adaptation
Source: Front Plant Sci. 2023 Nov 27;14:1260429. doi: 10.3389/fpls.2023.1260429 (PMC10712242; doi:10.3389/fpls.2023.1260429)
Supplement: Supplementary file 3 [file DataSheet_3.pdf]

Sample Preparation Method from User

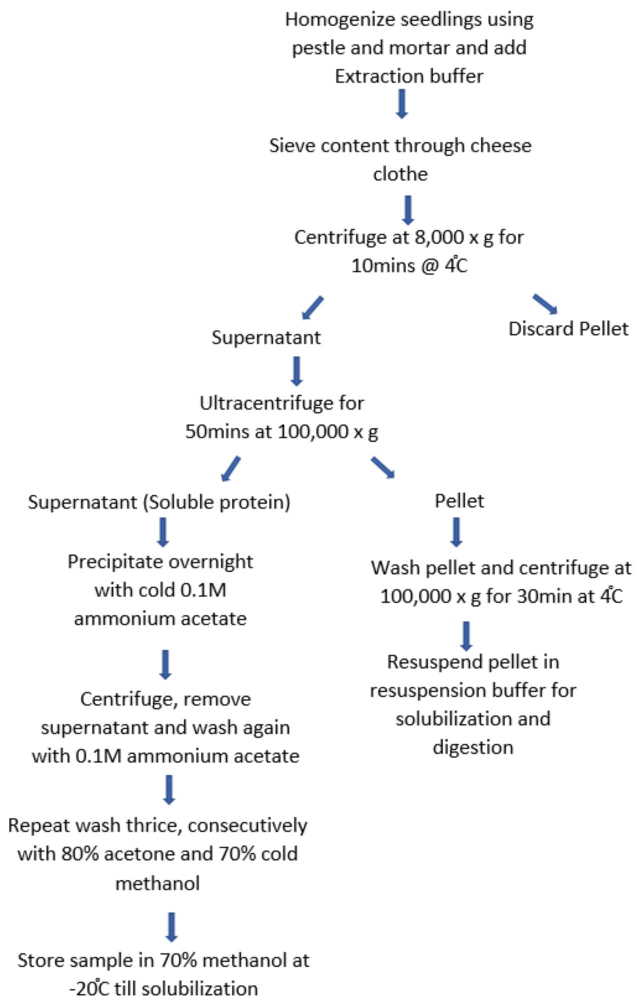

Sample Descriptions

Soluble proteins –large white pellets of precipitated protein in methanol stored at -20C

Membrane proteins – in Microsome Resuspension Buffer: 10 mM BTP–MES pH 7.8, 0.33 M sucrose, 3 mM KCl, and 5 mM potassium phosphate, pH 7.8 containing AEBSF (4-(2-Aminoethyl)benzenesulfonyl fluoride hydrochloride) – green solutions.

Membrane Protein Digestion

The membrane solutions were checked for protein content by SDS-PAGE. This shows that 15uL is ~5-10ug but with a large single band present (later found to be BSA).

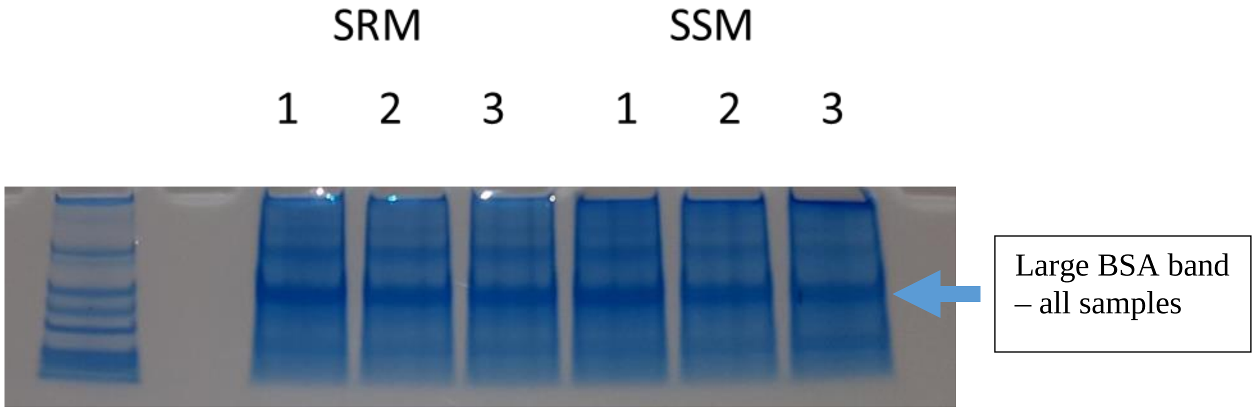

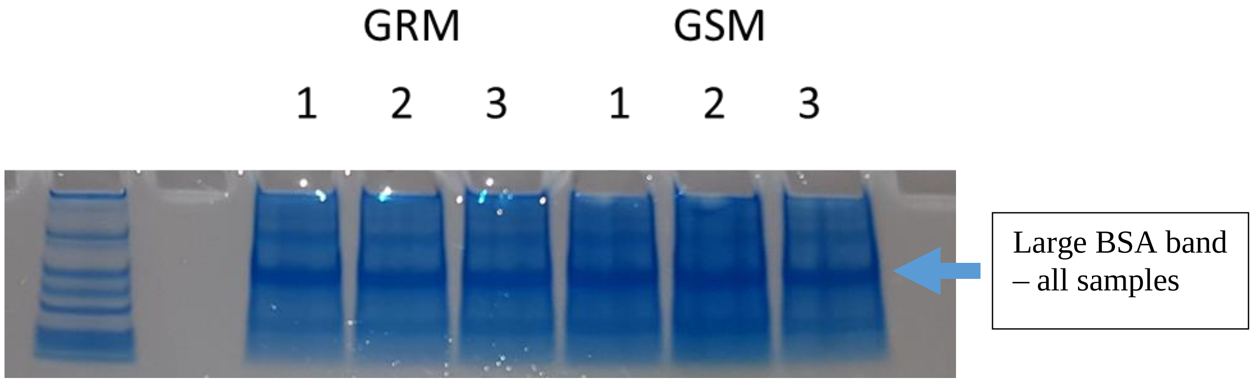

The membrane solutions were also assayed for protein content using the CB-X protein assay from G-Bioscience.

Sample ID	µg/20µL	Protein Type	Tissue Type
SR1M	9.9	Membrane	Root
SR2M	9.3	Membrane	Root
SR3M	9.7	Membrane	Root
SS1M	10.3	Membrane	Shoot
SS2M	8.7	Membrane	Shoot

SS3M	8.3	Membrane	Shoot
GR1M	8.5	Membrane	Root
GR2M	10.1	Membrane	Root
GR3M	9.6	Membrane	Root
GS1M	12.5	Membrane	Shoot
GS2M	12.5	Membrane	Shoot
GS3M	9.7	Membrane	Shoot

100ug of each membrane solution sample was made up to 0.5% sodium deoxycholate (SDC) and 5mM dithiothreitol (DTT) by using a 10% stock of SDC. Reduction was carried out at 37C for 3h before alkylation with 15mM iodoacetamide (IAM) for 45min at room temperature and subsequent quenching of the IAM with equimolar DTT. The pH was maintained at 7.8. Digestion was carried out for 24h at 37C by the addition of 4ug of Lys-C, then 2 x trypsin digestion with a 1:60 ratio of enzyme:substrate each for 16h. SDC was precipitated by the addition of trifluoroacetic acid and incubated at room temperature for 30min before removal by centrifugation at 16000 x g for 15min. This was repeated twice with fresh tubes.

Soluble Protein Digestion

The pelleted material was redissolved in a cocktail of 2M thiourea, 7M urea, 5mM DTT, 0.2M EPPS, pH 8.5. The solutions were checked for protein content using the CB-X protein assay from G-Bioscience and 200ug in 40uL was taken for digestion. Reduction was carried out at 37C for 3h before alkylation with 15mM iodoacetamide (IAM) for 45min at room temperature and subsequent quenching of the IAM with equimolar DTT. The urea cocktail was diluted to 3M with respect to the urea component and Lys-C (4ug) was added. Digestion was carried out for 16h at 37C. A further two aliquots of trypsin (3ug each) were added and digestion carried out for a total of 24h. Digestion was stopped by the addition of TFA to give a pH of 2.5.

CB-X Assay

Sample ID	µg/5µL	Protein Type	Tissue Type
SR1S	25.3	Soluble	Root
SR2S	27	Soluble	Root
SR3S	25.9	Soluble	Root
SS1S	27.4	Soluble	Shoot
SS2S	27.9	Soluble	Shoot
SS3S	29.3	Soluble	Shoot
GR1S	38.2	Soluble	Root
GR2S	36.1	Soluble	Root
GR3S	34.5	Soluble	Root
GS1S	38.4	Soluble	Shoot
GS2S	39.1	Soluble	Shoot
GS3S	35.3	Soluble	Shoot

TMT-labeling and bRPLC with subfractionation

All digests (soluble and membrane samples) were extensively desalted using 50mg Sep-Pak® C18 reverse-phase SPE columns (Waters Corp, Milford, MA). Eluted samples were dried down and redissolved in 100uL 200mM EPPS, pH 8.5. Six samples for each set were labeled using the TMT-6-plex reagent (ThermoFisher Scientific). For each sample, 100 µg of desalted peptides were labeled with 800 µg of TMT-6-plex reagent dissolved in 41uL of anhydrous acetonitrile. The labeled samples were combined into 4 6-plex (126, 127, 128, 129, 130, 131) experiments, acidified with trifluoroacetic acid to pH 2.5 and desalted using 50mg Sep-Pak® C18 reverse-phase SPE columns (Waters Corp, Milford, MA). 300 µg of each set was sub-fractionated offline into 96 fractions using high pH reverse phase C18 chromatography (ACQUITY UPLC® BEH™ C18, 1.7µm, 2.1 x 100mm, Waters Corp) at pH 10 and then fractions from t=15 to t=95 min were recombined to give a total of 6 fractions according to the strategy of Yang et al (2012).

REF: *Yang F, Shen Y, Camp DG 2nd, Smith RD. High-pH reversed-phase chromatography with fraction concatenation for 2D proteomic analysis. Expert Rev Proteomics. 2012 Apr;9(2):129-34.*

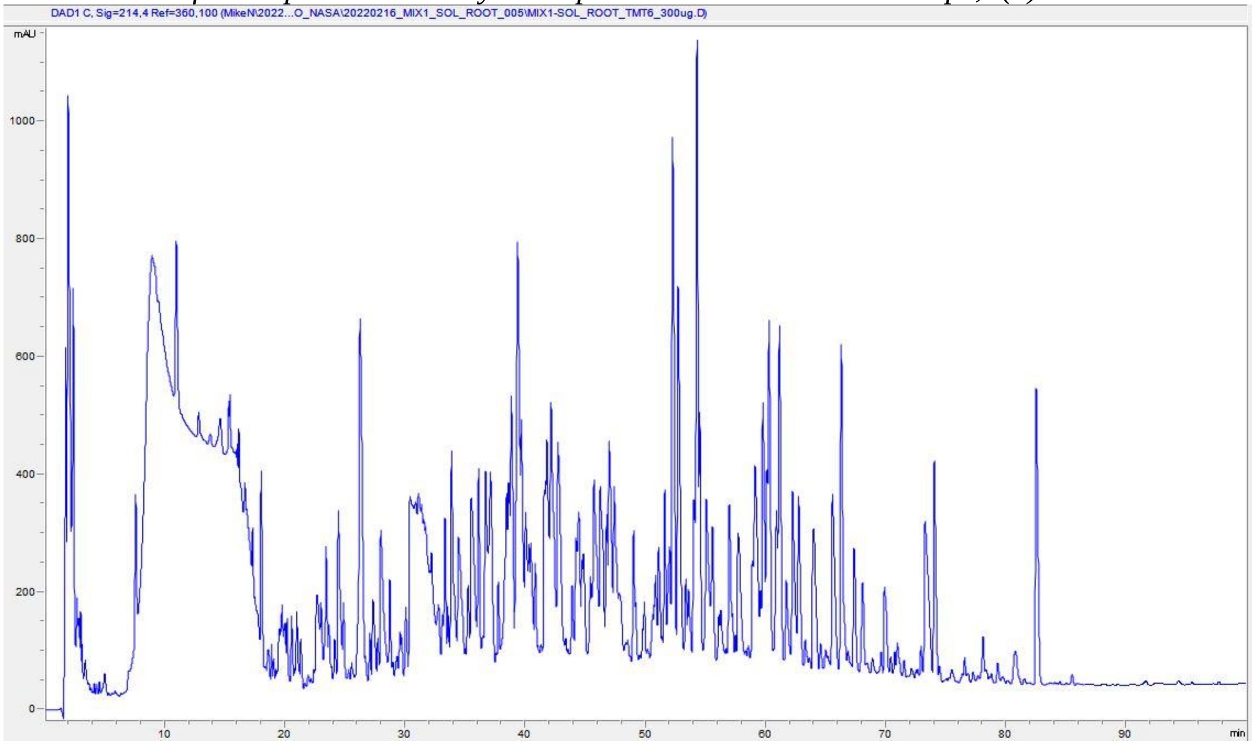

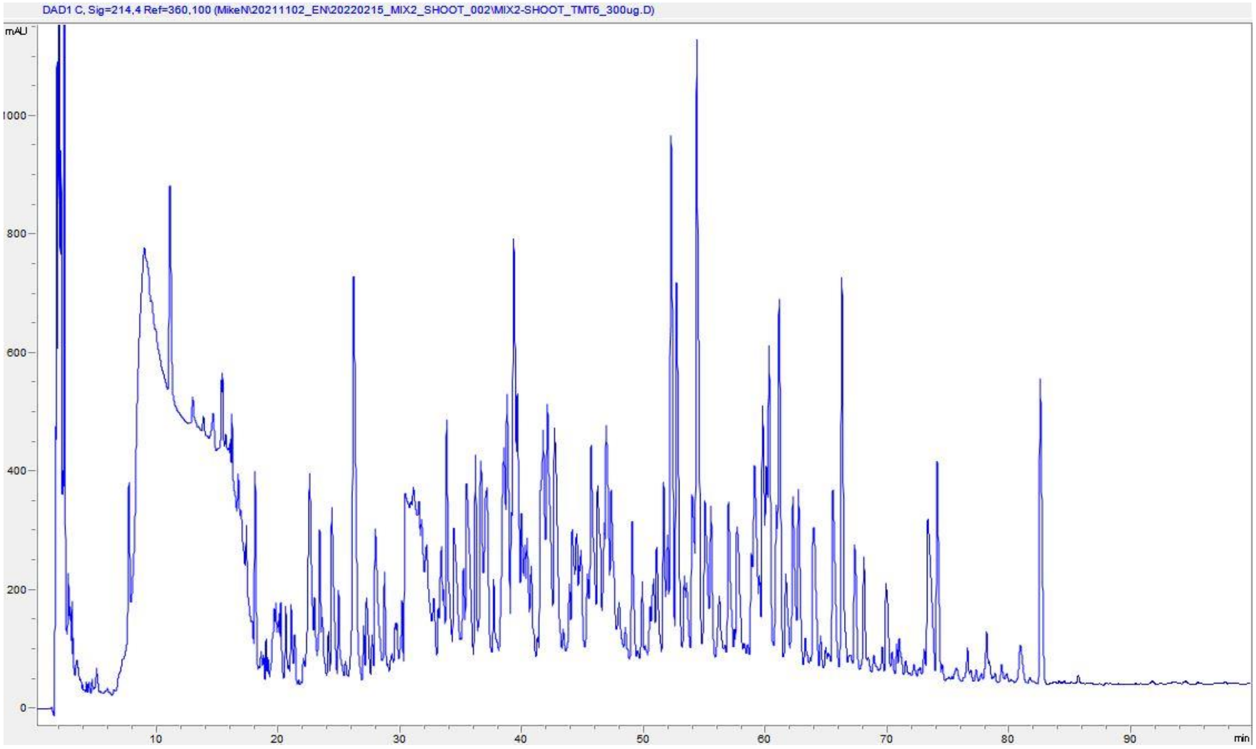

DAD1 C, Sig=214,4 Ref=360,100 (MikeN\20211102_EN\20220215_MIX2_SHOOT_002\MIX2-SHOOT_TMT6_300ug.D)
mAU
1000
800
600
400
200
0
10
20
30
40
50
60
70
80
90
min

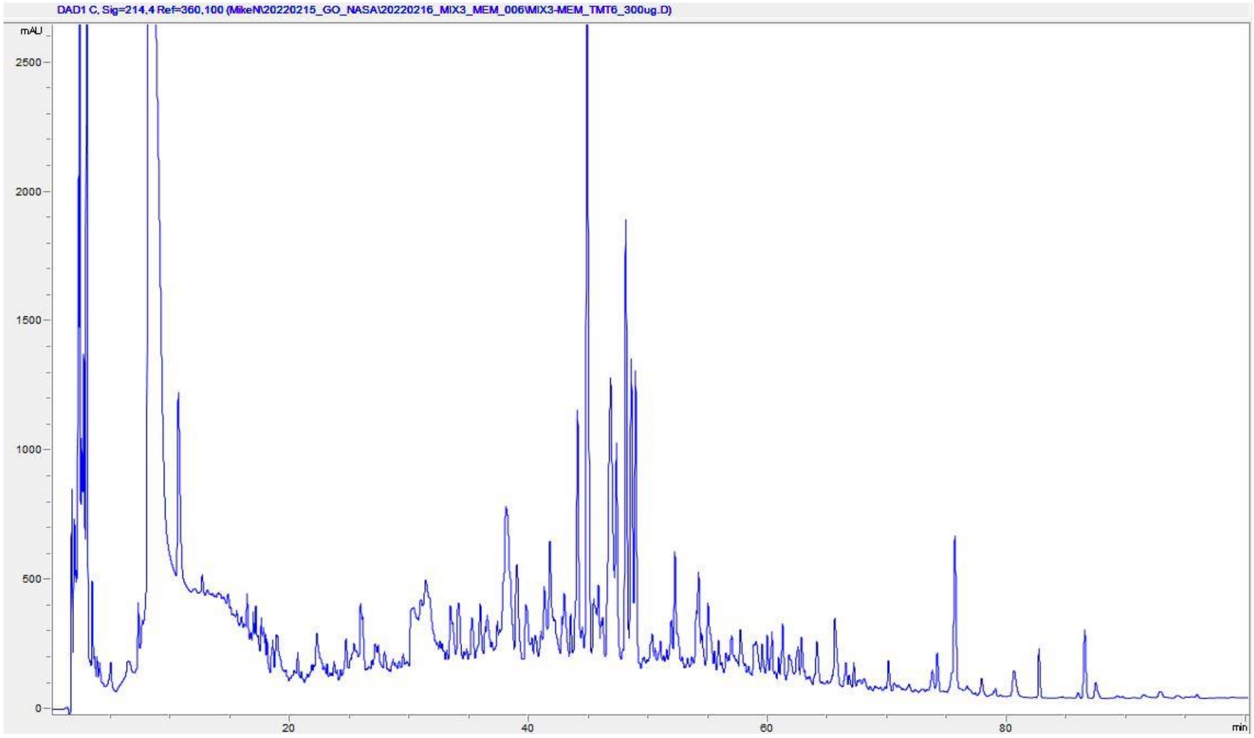

DAD1 C, Sig=214,4 Ref=360,100 (MikeN\20220215_GO_NASA\20220216_MIX3_MEM_006\MIX3-MEM_TMT6_300ug.D)
mAU
2500
2000
1500
1000
500
0
20
40
60
80
min

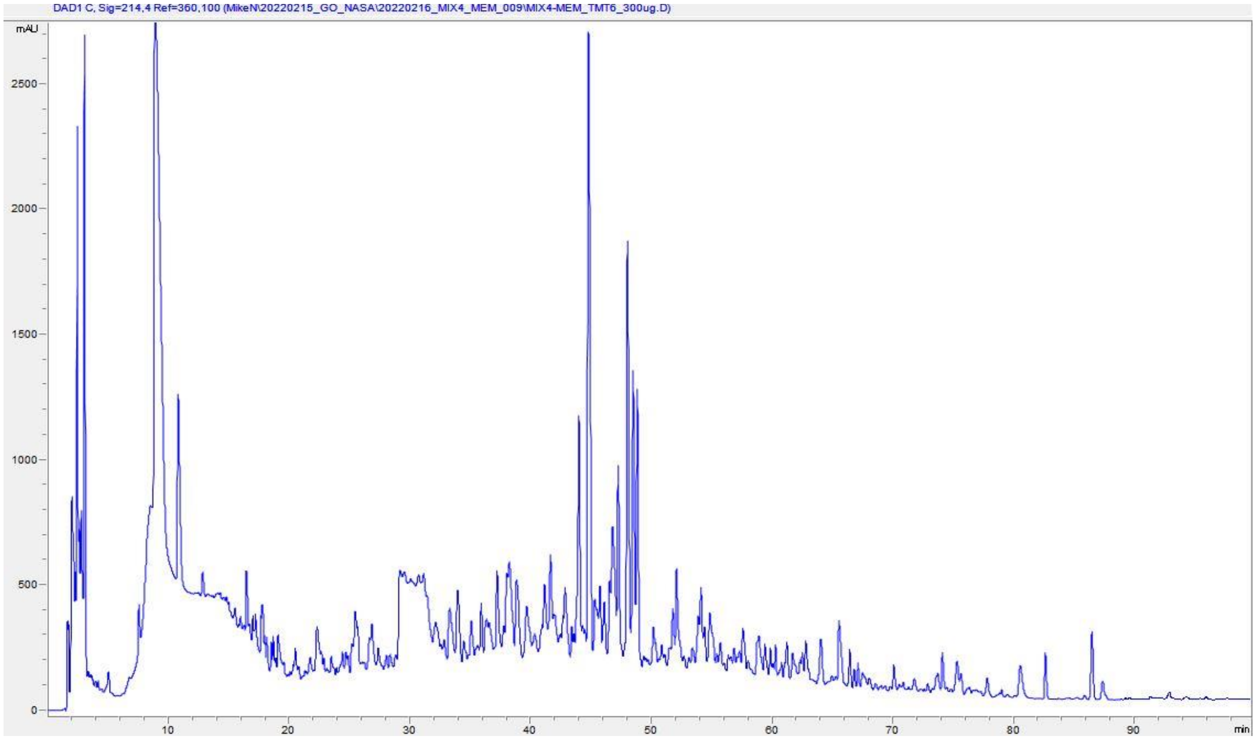

LC-MS/MS analysis

Each of the 6 fractions was analysed by LC-MS/MS on an RSLCnano system (ThermoFisher Scientific) coupled to an Eclipse Orbitrap Tribrid mass spectrometer (ThermoFisher Scientific). The samples were first injected onto a trap column (Acclaim PepMap™ 100, 75µm x 2 cm, ThermoFisher Scientific) for 3.0 min at a flow rate of 5 µL/min, 1% acetonitrile, 0.1% trifluoroacetic acid before switching in-line with the main column. Separation was performed on a C18 nano column (Acquity UPLC® M-class, Peptide CSH™ 130A, 1.7µm 75µm x 250mm, Waters Corp) at 300 nL/min with a linear gradient from 4-22% over 2 h. The LC aqueous mobile phase contained 0.1% (v/v) formic acid in water and the organic mobile phase contained 0.1% (v/v) formic acid in 100% (v/v) acetonitrile. Mass spectra for the eluted peptides were acquired on an Eclipse mass spectrometer using Synchronous Precursor Selection to allow accurate quantification of reporter ion ratios in MS3 scans. Real-time search (RTS) mode was switched on to limit selection of precursors to just *Arabidopsis* peptides (with fixed modifications of carbamidomethylation of cysteines and TMT-6plex of lysines and variable modification of oxidized methionines) for MS3 fragmentation. This avoided analysis of the high levels of contaminating BSA. High Field Asymmetric Waveform Ion Mobility Spectrometry (FAIMS Pro™ interface) was also employed to further subfractionate peptide species for deeper coverage. Both FAIMS and SPS help alleviate the negative influence of interfering ions and the ratio compression issues associated with TMT labeling.

Data analysis

Data were analyzed in Proteome Discoverer 2.4 software (ThermoFisher Scientific) using the Sequest HT search engine. Methionine oxidation was set as a variable modification, whilst TMT-6plex (K) and TMT-6plex (N-term) and cysteine carbamidomethylation were specified as fixed modifications. A maximum of 1 trypsin missed cleavages was permitted and the precursor and fragment mass tolerances were set to 10 ppm and 0.06 Da, respectively. Peptides were validated by Percolator with a 0.01 posterior error probability (PEP) threshold. The data were searched using a decoy database to

set the false discovery rate to 1% (high confidence). The protein quantification was processed using the co-isolation threshold set to 50% and the average S/N to 10. The peptides were quantified using the peak intensity of the reporter ion in the MS2 spectrum. The peak abundance was normalized for differences in sample loading using total peptide amount where the peptide group abundances are summed for each sample and the maximum sum across all runs is determined. The pooled sample was used to normalize each TMT experiment based on the same sample. The protein ratios and log2 fold change are calculated from the grouped protein abundances. The significance of differential expression is tested using an ANOVA test which provides a p-value and an adjusted p-value using the Benjamini-Hochberg method for all the calculated ratios.
